# A Conformation Selective Mode of Inhibiting SRC Improves Drug Efficacy and Tolerability

**DOI:** 10.1158/0008-5472.CAN-21-0613

**Published:** 2021-08-20

**Authors:** Carolin Temps, Daniel Lietha, Emily R. Webb, Xue-Feng Li, John C. Dawson, Morwenna Muir, Kenneth G. Macleod, Teresa Valero, Alison F. Munro, Rafael Contreras-Montoya, Juan R. Luque-Ortega, Craig Fraser, Henry Beetham, Christina Schoenherr, Maria Lopalco, Mark J. Arends, Margaret C. Frame, Bin-Zhi Qian, Valerie G. Brunton, Neil O. Carragher, Asier Unciti-Broceta

**Affiliations:** 1 Cancer Research UK Edinburgh Centre, Institute of Genetics & Cancer, University of Edinburgh, Edinburgh, United Kingdom.; 2 Margarita Salas Center for Biological Research (CIB), Spanish National Research Council (CSIC), Madrid, Spain.; 3 MRC Centre for Reproductive Health, University of Edinburgh, Edinburgh, United Kingdom.; 4 Edinburgh Innovations Ltd., Edinburgh, United Kingdom.

## Abstract

Despite the approval of several multikinase inhibitors that target SRC and the overwhelming evidence of the role of SRC in the progression and resistance mechanisms of many solid malignancies, inhibition of its kinase activity has thus far failed to improve patient outcomes. Here we report the small molecule eCF506 locks SRC in its native inactive conformation, thereby inhibiting both enzymatic and scaffolding functions that prevent phosphorylation and complex formation with its partner FAK. This mechanism of action resulted in highly potent and selective pathway inhibition in culture and *in vivo*. Treatment with eCF506 resulted in increased antitumor efficacy and tolerability in syngeneic murine cancer models, demonstrating significant therapeutic advantages over existing SRC/ABL inhibitors. Therefore, this mode of inhibiting SRC could lead to improved treatment of SRC-associated disorders.

**Significance:**

Small molecule–mediated inhibition of SRC impairing both catalytic and scaffolding functions confers increased anticancer properties and tolerability compared with other SRC/ABL inhibitors.

## Introduction

The SRC kinase, product of the first cellular proto-oncogene identified (1–6), emerged as a potential therapeutic target in the early 1980s (7). Although decades of research have assisted in elucidating the central role of this nonreceptor tyrosine kinase in the transduction of multiple oncogenic signals, either via receptor tyrosine kinases, cell-to-cell or extracellular matrix-to-cell communications (7–12), accumulating evidence of additional roles for SRC in cancer keeps it in the spotlight (13–16). Predominantly inactive in noncancerous cells, SRC is aberrantly activated in many cancer types, including breast, colon, prostate, pancreatic, and ovarian cancers (17). With few exceptions (18), increased SRC activity is generally associated with late-stage cancers, metastatic potential, and resistance to therapies, and correlates with poor clinical prognosis (19–21). Surprisingly, despite the vast amount of evidence gathered over the years and the approval of dual SRC/ABL kinase inhibitors to treat *BCR-ABL*-positive leukemias (22), to date no kinase inhibitor has yet been approved for the treatment of SRC-active solid malignancies.

Because of high structural similarities between their active sites (23), most small molecule inhibitors targeting SRC family kinases—including the clinical drugs dasatinib and bosutinib—display equal potency against the nonreceptor tyrosine kinase ABL. Although dual inhibition of ABL and SRC can be beneficial in the treatment of hematologic cancers (24), this polypharmacologic profile is not desirable to treat other tumors, as several studies have found that ABL kinase can act as a tumor suppressor in different malignancies (25–31). Moreover, ABL regulates cardiomyocyte growth and development (32), and its inhibition is directly linked to cardiotoxic events (33, 34) and immunosuppression (35) in patients with leukemia, which makes unwanted ABL inhibition a clinical liability.

**Figure 1.**
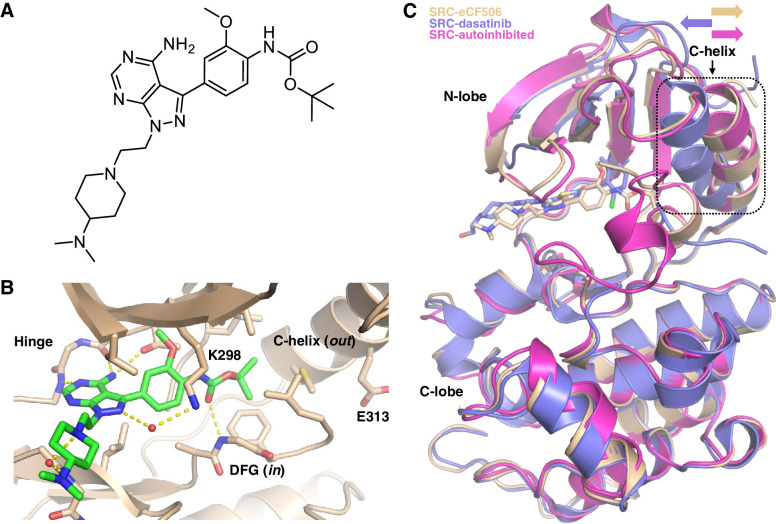
**A,** Chemical structure of eCF506. **B,** Structure of eCF506 bound to the inactive conformation of the SRC kinase domain (DFG *in*, C-helix *out*). Amino acid numbering corresponds to human SRC. **C,** Superimposed structures of autoinhibited SRC kinase (PDB 2SRC) and co-crystal structures SRC:eCF506 (PDB 7NG7) and SRC:dasatinib (PDB 3G5D). The orientation of the C-helix is highlighted.

The novel SRC inhibitor eCF506 (Fig. 1A) displays subnanomolar activity against SRC (36), a potency that is on par with the current best-in-class SRC/ABL inhibitor dasatinib (37). In contrast, eCF506 requires concentrations three orders of magnitude greater to inhibit ABL, which differentiates it from existing SRC/ABL inhibitors. As the reasons behind this selectivity could be beneficial for the treatment of SRC-associated pathologies, we set to investigate the mode of binding of eCF506 to SRC and the molecular phenotype of eCF506-treated breast cancer cells compared with clinical SRC/ABL inhibitors. Herein, we report compelling structural and mechanistic data that defines the mode of SRC inhibition by eCF506, which provides explanation to the selectivity and potency of this promising drug candidate. Using syngeneic models of breast cancer and bone metastasis, we also show that the unique properties of eCF506 translates into increased antitumor efficacy and tolerability.

## Materials and Methods

### Chemicals

eCF506 was synthesized in five steps from commercially available starting materials following the procedure previously reported (36). Dasatinib was acquired from Cell Guidance Systems (SM45). Bosutinib (S1014), saracatinib (S1006), and imatinib (S2475) were purchased from Selleck Chemicals. Citrate buffer preparation: 18 mL of 0.1 M citric acid (21 g in 1 L water), 82 mL of 0.1 sodium citrate (29.4 g in 1 L water), make up to 1 L with water.

### Cell lines

Cell lines were purchased from the European Collection of Authenticated Cell Cultures (MDA-MB-231 and MCF7) or were a kind donation from the cell bank of the Institute of Genetics and Cancer, Edinburgh. MetBo2 cells were established in Dr. Qian's lab. Cell lines in culture were tested for mycoplasma every 2 months and authenticated by STR profiling. Cells (at passage 10 or below) were grown in their specific culture medium (see Supplementary Materials and Methods) and maintained in a Heracell 240i tissue culture incubator (37°C, humidified air, 5% CO_2_).

### Coimmunoprecipitation

Cells were seeded in 10 cm dishes, left to attach overnight and treated with compounds for 6 hours (3 hours for FAK-IP) prior to lysis with RIPA buffer. Protein concentration was determined using a BCA assay and 1 mg/mL samples were prepared in a microcentrifuge tube, to which, 20 μL Dynabeads Magnetic Beads Protein A (Invitrogen, 10002D) were added. Two micrograms of primary SRC antibody (Cell Signaling Technology, 2109) or appropriate IgG control (Cell Signaling Technology, 2729) was added, and samples rotated at 4°C overnight. Dynabeads were washed twice with RIPA lysis buffer and three times with PBS before being resuspended in 20 μL of 1× Laemmli sample buffer. Samples were heated to 95°C for 5 minutes and beads were separated using the magnetic rack before lysates were analyzed for SRC (SCB, SC-8056) and FAK (Cell Signaling Technology, 3285) by standard Western blot analysis. For the FAK-IP 1 mg of cell lysate was incubated with anti-FAK (clone 4.47)–conjugated agarose antibody (Millipore, 5–537). The agarose beads were washed twice with RIPA buffer and twice with PBS before being resuspended in 20 μL of 1× Laemmli sample buffer. Samples were heated to 95°C for 5 minutes and analyzed for SRC (Cell Signaling Technology, 2109) and FAK (Cell Signaling Technology, 3285) by the Western blot analysis.

### Cell proliferation assays

Cells were seeded in 96-well plates, with edge wells being filled with PBS instead. After 48 hours, the medium was removed and 95 μL of fresh medium was added. Dilution plates of the test compounds were prepared in DMSO and diluted 1:50 into medium, of which, 5 μL were added to the cell plates (final DMSO concentration 0.1% v/v). Cells were treated for 5 days before cell viability was measured using PrestoBlue (Invitrogen). PrestoBlue was added to an untreated plate on day 2 and to the treated plates on day 7 (1 in 10 dilution) and fluorescence (550 nm excitation, 580 nm emission) was measured after the same incubation time on both days (0.25–6 hours after addition, depending on cell line) using a Perkin Elmer Envision 2101 plate reader. The average fluorescence on day 2 was subtracted from the values on day 7 to distinguish between cytotoxic and cytostatic treatment effects and the data were normalized to DMSO controls. GraphPad Prism 7 was used to fit nonlinear regression curves and determine the concentration for 50% growth inhibition (GI_50_).

### Western blotting

Cells were seeded in 6-well plates and incubated for 48 hours until approximately 70% confluence was reached. Compounds were added (final DMSO concentration 0.1% v/v) and cells incubated for the time specified (3 or 24 hours). Plates were put on ice, the medium was removed, and cells were washed twice with ice-cold PBS. RIPA lysis buffer with protease and phosphatase inhibitors was added (1.25 mmol/L PMSF, 0.1% v/v aprotinin, 100 μmol/L Na_3_VO_4_, 500 μmol/L NaF) and the cells were scraped, collected, and incubated on ice for 10 minutes with occasional vortexing. Lysates were centrifuged at 4°C (17,000 × *g*, 10 min) and the supernatant was transferred into a new microcentrifuge tube. The Pierce BCA Protein Assay Kit (Thermo Fisher Scientific, 23225) was used to determine the protein concentration according to the manufacturer's instructions, with samples being diluted one in four and set up in duplicate on 96-well plates. Plates were read on a Spark 20M microplate reader (Tecan) and absorbance was measured at 562 nm. Samples for SDS-PAGE were prepared as follows: 20 μg protein was diluted with RIPA buffer to a final volume of 15 μL, to which, 5 μL of 4X Laemmli Sample Buffer (Bio-Rad, 1610747) were added. Samples were heated to 95°C for 5 minutes and then loaded onto 12-well 4–15% Mini-PROTEAN TGX precast gels (Bio-Rad, 4561045). Precision Plus Protein Dual Color Standards (Bio-Rad, 1610374) were loaded alongside the lysates. Gels were run in Mini-PROTEAN Tetra Cell tanks in TGS buffer (pH 8.3) at 150 V for 45 minutes. Proteins were transferred onto Trans-Blot Turbo Nitrocellulose membranes (Bio-Rad, 1704159) using the Trans-Blot Turbo Transfer System (Bio-Rad) for semi-dry blotting at 2.5 A (midi) or 1.3 A (mini) and up to 25 V for 10 minutes. Membranes were cut and blocked with blocking buffer (TBST 0.1% with 5% w/v BSA) for 1 hour at room temperature before incubation with primary antibodies at 4°C overnight. Membranes were washed twice with TBST (0.1%) for 3 minutes before addition of secondary antibodies and incubation for 2 hours at room temperature, before washing another three times with TBST (0.1%) for 5 minutes. Antibodies were detected by chemiluminescence by incubating membranes for 2 minutes with the Clarity ECL Western Blotting Substrates (Bio-Rad, 102031309) before imaging in the ChemiDoc XRS+ Imaging System (Bio-Rad). Protein bands were quantified using the ImageLab software v5.2 (Bio-Rad) with automated band detection and background subtraction (disk size 70 mm).

### Animal experiments

Animal experiments were performed under Home Office License in compliance with the Animals (Scientific Procedures) Act 1986 and approved by the University of Edinburgh Ethics Committee.

#### Bone metastasis model

FVB/NHanHSD (FVB) mice were anesthetized by isoflurane and injected with 100,000 MetBo2 cells into the left ventricle. Tumor growth was monitored by bioluminescence imaging on the Optima imager (Biospace). Bone metastasis tumors were allowed to grow for 7 days until the signals of bone metastasis were clearly detected by Optima imager. Eleven mice that developed tumors in the hind legs or jaw were included in the study. Mice were randomized and put into control (5 mice) or treatment (6 mice) groups. Treated mice received daily eCF506 by oral gavage (40 mg/kg in ultrapure water, dissolved at 8 mg/mL) and control group mice received vehicle only. Mice were imaged twice a week and the bioluminescence intensity of tumors in the hind legs or jaw was quantified by M3 software. Mice were culled when the tumor burden became too great.

#### Orthotopic breast cancer model

FVB or CD1 nude mice were anesthetized by isoflurane and implanted in the left fourth mammary fat pad with one million MetBo2 cells. Tumors were allowed to grow until they reached around 50 mm^3^ (FVB) or 40 mm^3^ (CD1) in size (100 mm^3^ for the eCF506 versus dasatinib study), after which, mice were randomly allocated into two groups. Mice were treated with vehicle (3 mmol/L sodium citrate buffer pH 3.0) or eCF506 (40 mg/kg, dissolved at 4 mg/mL in vehicle) once daily by oral gavage (100 μL per 10 g mouse weight) for 3 days (PD study) or 28 days (efficacy study). Of note, the use of citrate buffer instead of ultrapure water allowed the production of a clear solution of eCF506 just by mixing. Tumor size was measured by caliper and mice weighed twice a week. Mice were culled by cervical dislocation once tumor size reached 15 mm in diameter. After the initial treatment phase, surviving mice were monitored until tumors relapsed. For the eCF506 versus dasatinib study, treatment restarted once average tumor size reached approximately 0.3 to 0.4 cm^3^. Mouse that showed no tumor regrowth were monitored (tumor volume and animal weight) during the duration of the study. Mice were dosed 2 hours prior to culling by cervical dislocation, after which, tumors were removed and fixed in neutral buffered formalin for IHC and hematoxylin and eosin (H&E) staining. For the vehicle versus dasatanib (20 mg/kg) versus eCF506 study (40 mg/kg), mice were randomized into groups and treatment initiated as above once tumors were detectable. Treatment was ceased at day 28, with treatment being resumed for individual mice once increasing tumor growth was observed. Once tumor endpoint (15 mm diameter) was reached, mice were culled and tumors, spleens, and hearts of mice were dissected and weights were recorded. Tissues were fixed in neutral buffered formalin and paraffin embedded for IHC and H&E analysis.

## Results

### eCF506 targets the native inactive conformation of SRC

The majority of SRC kinase inhibitors bind to the catalytic domain of SRC in its active conformation (38). To characterize the binding mode of eCF506, we co-crystalized human SRC (kinase domain) bound to eCF506 and determined the structure to 1.5 Å (for crystallographic and refinement statistics see Supplementary Table S1, Supplementary Materials and Methods). Remarkably, the structure revealed that, contrary to our previous prediction (36), eCF506 binds to the native inactive conformation of the SRC kinase (Fig. 1B and C). In this conformation, the C-helix is moved out and the K298-E313 salt bridge (a hallmark of active kinases) is not formed (Fig. 1B; Supplementary Figs. S1A and S1B, Supplementary Materials and Methods), thereby switching kinase activity off.

**Figure 2.**
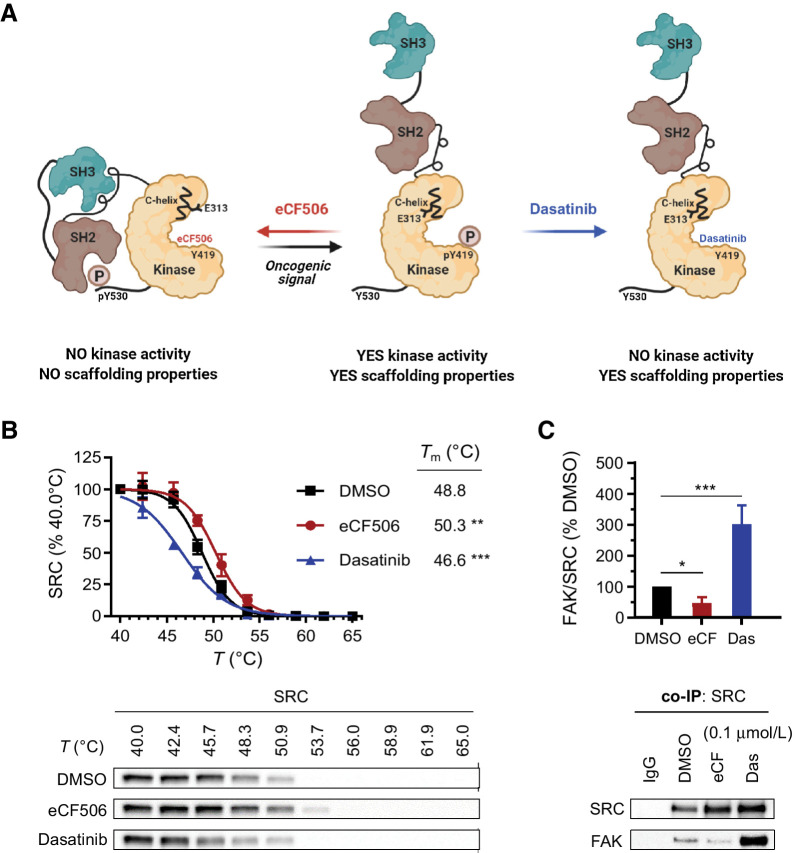
**A,** Proposed structural changes induced by eCF506 and dasatinib on human SRC protein. Created with BioRender. **B,** Thermal shift assay. MDA-MB-231 cells were treated with eCF506 (0.3 μmol/L), dasatinib (0.3 μmol/L), or DMSO for 1 hour prior to heating, cell lysis and analysis of SRC by Western blot anaysis. Band intensities were normalized to the lowest temperature band for each condition. Error bars, ±SD (*n* = 3). **, *P* < 0.01; ***, *P* < 0.001 (ANOVA). Bottom, representative Western blots are shown. **C,** Coimmunoprecipitation (co-IP) study in MDA-MB-231 cells. Cells were treated with eCF506 (0.1 μmol/L), dasatinib (0.1 μmol/L), or DMSO for 6 hours and lysates incubated overnight with magnetic beads functionalized with anti-SRC IgG, followed by bead separation and analysis of SRC and FAK by Western blot analysis. Band intensities were normalized to SRC and compared with DMSO values. Error bars, ±SD (*n* = 3). *, *P* < 0.05 (*t* test); ***, *P* < 0.001 (ANOVA). Bottom, representative Western blots.

In the native autoinhibited conformation of SRC, the SH2 and SH3 domains interact with the kinase domain and form a compact structure that blocks macromolecular assemblies with other proteins. In contrast, these domains are released in the active state of SRC and the protein adopts an open conformation that is free to interact with other proteins (Fig. 2A), including FAK at focal adhesions (39). Biophysical characterization of the hydrodynamic radius of full length SRC (human SRC 84–536) in the absence (Apo) and presence of dasatinib or eCF506 show that, whereas dasatinib induces opening of the SRC structure, eCF506 maintains SRC in a more compact conformation, similar to the Apo form (Supplementary Table S2, Supplementary Materials and Methods). Consistent with these studies, a thermal shift assay determined that eCF506 and dasatinib induce significant but opposite effects on apparent melting temperature (*T*_m_) of SRC compared with DMSO, with the former stabilizing the protein but the latter destabilizing it (Fig. 2B).

### eCF506 inhibits the formation of the SRC–FAK complex

The binding mode of eCF506, by which the compound locks SRC into a closed inactive conformation, implies that upon eCF506 binding the SH3 and SH2 domains will not be exposed to physically interact with other proteins (Fig. 2A). Hence, we predicted that the capacity of SRC to form complexes with its binding partner FAK, one of its key substrates, should be reduced. This hypothesis infers in consequence the opposite effect for classical kinase inhibitors that block SRC in its active conformation, that is, the capacity of SRC to assemble with other proteins should be enhanced. To test this, we studied SRC–FAK binding in breast cancer MDA-MB-231 cells by coimmunoprecipitation after treatment with eCF506 and clinical kinase inhibitors dasatinib, bosutinib, and saracatinib. As seen in Fig. 2C, dasatinib treatment increased by 3-fold the levels of FAK in complex with SRC relative to the DMSO control, whereas eCF506 decreased the recovery of FAK protein by 50%. Bosutinib and saracatinib also increased binding of SRC to FAK (Supplementary Fig. S2A, Supplementary Materials and Methods). Consistent with these findings, coimmunoprecipitation studies with magnetic beads functionalized with anti-FAK IgG led to equivalent results, that is, dasatinib but not eCF506 increased SRC levels co-precipitating with FAK relative to the untreated control (Supplementary Fig. S2B, Supplementary Materials and Methods). These findings fully agree with the proposed mode of inhibition of SRC by eCF506.

### eCF506 displays high selectivity across the kinome

Previous investigations (36) had only tested the activity of eCF506 in a small panel of kinases (see Supplementary Table S3). To determine the kinome-wide activity profile of eCF506, an enzymatic inhibition screen was carried out with eCF506 at 1 μmol/L against 340 wild-type protein kinases (*n* = 2) by Reaction Biology Corp. With 0.1% of remnant enzymatic activity, the highest degree of inhibition achieved by eCF506 across the whole kinase panel was to the protein SRC. From the 340 kinases tested, only 25 of them decreased their activity below 50% (see full list in Supplementary Table S4, Supplementary Materials and Methods). This compares favorably with clinical SRC/ABL inhibitors dasatinib and bosutinib, where the number of hit kinases is 51 and 85, respectively, using half of the concentration used for eCF506 (Supplementary Table S5, Supplementary Materials and Methods). In agreement with previous results (36), ABL inhibition by eCF506 was much lower than for dasatinib and bosutinib (56% ABL activity remains after treatment, vs. 2.8% and 1.8%, respectively) and nine of the identified hits are kinases from the SRC family, with levels of remnant enzymatic activity between 5% and 0.1%. The rest of the hits are members of the tyrosine kinase or tyrosine kinase-like family branches. Outside the SRC family, the most potent hit found in the screening was the nonreceptor tyrosine kinase BRK. However, determination of its IC_50_ value (see Supplementary Table S3) showed that the potency of eCF506 against SRC (IC_50_ < 0.0005 μmol/L) is significantly higher than against BRK (IC_50_ = 0.0173 μmol/L), with a 35-fold difference in activity between these targets.

### eCF506 exhibits lower antiproliferative activity than SRC/ABL inhibitors in *BCR-ABL*–positive leukemia cells

To study if the selectivity profile of eCF506 translate into weaker phenotypic activity against ABL-driven cancer cells relative to classical SRC/ABL inhibitors, we performed cell proliferation assays with three *BCR-ABL*-positive chronic myeloid leukemia (CML) cell lines: LAMA-84, KCL-22, and K-562. Inhibitors (eCF506, SRC/ABL inhibitors dasatinib, bosutinib, and saracatinib, and ABL inhibitor imatinib) were tested at a range of concentrations and their GI_50_ values calculated (see Supplementary Fig. S3, Supplementary Materials and Methods). Notably, results show that there is approximately 1,000-fold difference in activity between dasatinib and eCF506 in the cell lines tested, which correlates well with their difference in activity against ABL in biochemical assays (see Supplementary Table S3).

### eCF506 exhibits potent antiproliferative activity in ER^+^ and triple-negative breast cancer cells

**Figure 3.**
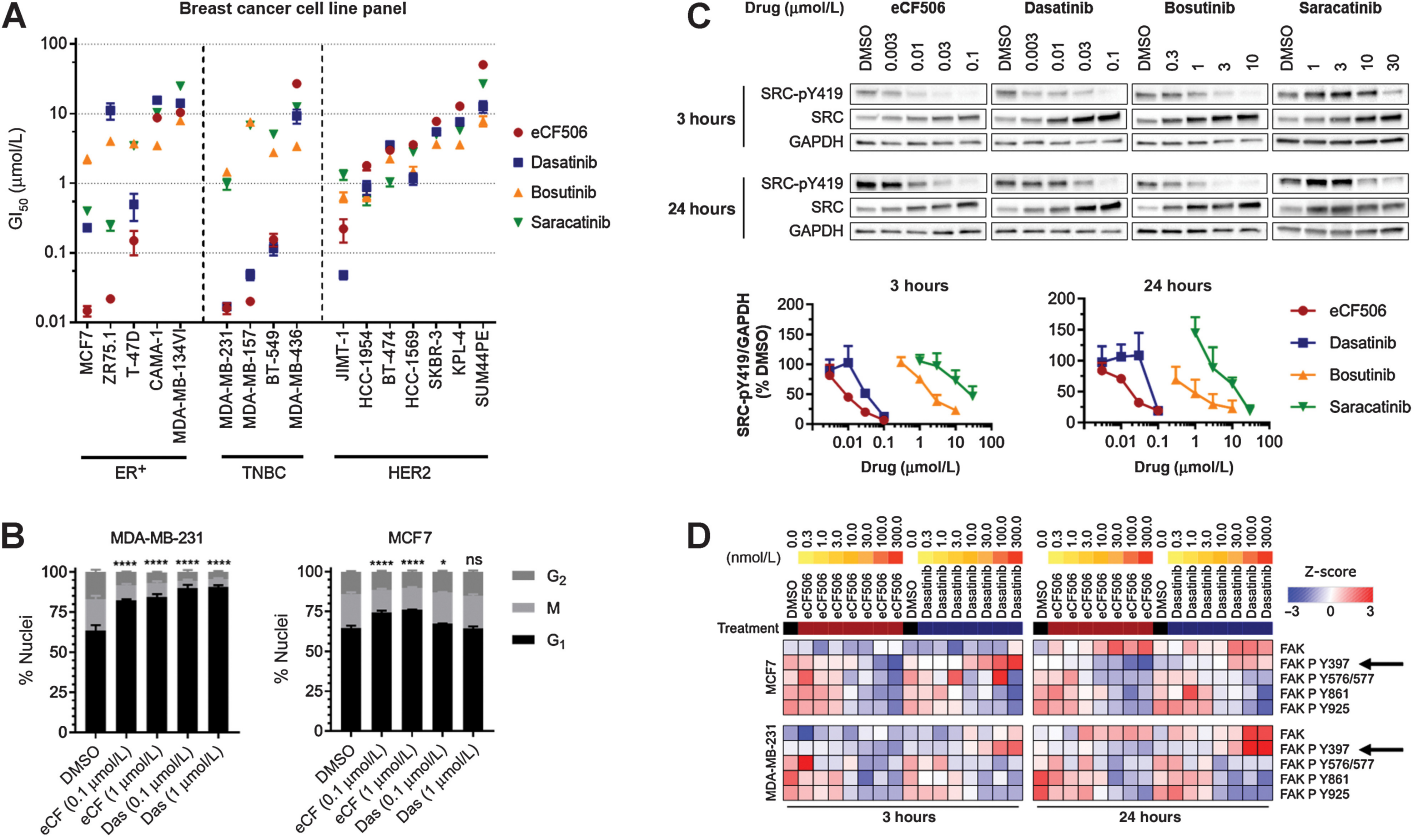
**A,** Antiproliferative activities of eCF506, dasatinib, bosutinib, and saracatinib across 16 breast cancer cell lines. Calculated GI_50_ values are ranked by sensitivity to eCF506. Error bars, ±SD (*n* = 3). **B,** Cell-cycle analysis after treating MDA-MB-231 and MCF7 cells with eCF506 or dasatinib for 48 hours. Error bars, ± SD (*n* = 3). Changes in G_1_ phase compared by one-way ANOVA (Dunnett correction for multiple comparison); *, *P* < 0.05; ****, *P* < 0.0001; ns, nonsignificant. **C,** Representative Western blots after treating MDA-MB-231 for 3 and 24 hours with eCF506, dasatinib, bosutinib, and saracatinib. Plots of normalized SRC-pY419 levels after each treatment are shown at the bottom. Error bars, ±SD (*n* = 3). **D,** Analysis of total and phosphorylated FAK from the RPPA study in MCF7 (top) and MDA-MB-231 (bottom) cells after 3 hours (left) and 24 hours (right) treatment with eCF506 and dasatinib at 0.3 to 300 nmol/L. Arrows, FAK-pY397.

Encouraged by the distinct mechanism of action and increased selectivity of eCF506, we screened eCF506 and clinical SRC/ABL inhibitors against a panel of 16 breast cancer cell lines. As before, cell viability was determined before and after adding the inhibitor to calculate the concentration needed to inhibit proliferation by 50% (GI_50_). The resulting GI_50_ values are ranked by cancer subtype and sensitivity to eCF506 in Fig. 3A (dose–response curves are provided in Supplementary Fig. S4, Supplementary Materials and Methods). Seven cell lines treated with eCF506 displayed low GI_50_ values (0.015–0.22 μmol/L): the triple negative cell lines BT-549, MDA-MB-157, and MDA-MB-231; the ER^+^ cell lines MCF7, ZR-75.1, and T-47D; and the HER2^+^ cell line JIMT-1. The potency of eCF506 in these cell lines was as high as or even higher (in ER^+^ cells) than that of dasatinib. On the contrary, with the exception of JIMT-1 (cell line with acquired resistance to anti-HER2 therapy; ref. 40), HER2^+^ breast cancer cell lines showed low to negligible reduction of cell proliferation after eCF506 treatment (GI_50_ > 1.8 μmol/L), which agrees with the expected minor role played by SRC in HER2^+^ breast cancer cells before becoming resistant to trastuzumab (41). Overall, eCF506 was the most potent inhibitor against triple negative and ER^+^ cell lines showing sensitivity (GI_50_ < 1 μmol/L) for SRC inhibitors, whereas displayed lower inhibition of cell proliferation against HER2^+^ cell lines than any other SRC inhibitor, further evidence of the high potency and selectivity, respectively, of eCF506.

Analysis of the dose–response curves shows that eCF506 displayed anti-proliferative (not cytotoxic) effects at submicromolar concentrations in all cell lines that exhibited sensitivity to treatment (Supplementary Fig. S4, Supplementary Materials and Methods). Cell-cycle assays in MDA-MB-231 and MCF7 cells confirmed that both eCF506 and dasatinib induce arrest in the G_1_-phase (Fig. 3B). In agreement with the cell viability assays, the cell-cycle arrest mediated by eCF506 in MCF7 cells was greater than that of dasatinib at the concentrations tested.

To test the selectivity of eCF506 on a model of nonmalignant breast epithelial cells, MCF10A cells were treated with eCF506 and dasatinib at a range of concentrations (0.001–10 μmol/L) and cell viability measured at day 5. As shown in Supplementary Fig. S5 (Supplementary Materials and Methods), eCF506 had minimal impact on cell proliferation in this noncancerous cell line (EC_50_ > 10 μmol/L), whereas dasatinib induced >50% reduction of cell viability at low micromolar levels.

### eCF506 inhibits cellular SRC activity more potently than clinical SRC/ABL inhibitors

SRC undergoes autophosphorylation of its kinase domain at Y419 upon activation by upstream signaling events (8, 9), thus serving as a direct readout of SRC kinase activity. As seen in Fig. 3C, eCF506 and dasatinib led to almost complete inhibition of SRC phosphorylation at 0.1 μmol/L in MDA-MB-231 cells, whereas eCF506 displayed superior potency at lower concentrations. Of note, eCF506 induced much more potent inhibition of SRC-pY419 compared with dasatinib in MCF7, T-47D, and ZR-75.1 cells (Supplementary Fig. S6, Supplementary Materials and Methods), which correlates with the superior antiproliferative activity of eCF506 in ER^+^ cell lines. On the other hand, both inhibitors led to complete SRC-pY419 inhibition in HER2^+^ JIMT-1 cells at 0.1 μmol/L, suggesting that the superior antiproliferative activity of dasatinib versus eCF506 in this cell line (GI_50_ = 0.048 and 0.222 μmol/L, respectively) is due to the effects of dasatinib on pathways that do not depend on SRC. Bosutinib and saracatinib displayed weaker inhibition of SRC autophosphorylation in all the cell lines tested, requiring micromolar concentrations to induce significant reduction of SRC-pY419 levels.

### eCF506 indirectly reduces FAK autophosphorylation

To evaluate the overall impact of eCF506 and dasatinib upon pathway signaling, we carried out a reverse phase protein array (RPPA) screen to identify differences in total protein levels and posttranslational modifications in MCF7 cells and MDA-MB-231 cells using the Zeptosens RPPA platform as described previously (42). As expected, downregulation of SRC-pY419 and SRC phosphorylation sites on FAK (Y576/577, Y861, Y925), and compensatory upregulation of total SRC was observed with both compounds, along with effects on a number of proteins involved in the cell cycle and survival signaling pathways (see complete heat maps and Western blot analysis of selected proteins in Supplementary Figs. S7A–S7D, Supplementary Materials and Methods). The most consistent and clear difference between dasatinib and eCF506 detected by RPPA was the differential phosphorylation of FAK-Y397 (Fig. 3D). Dasatinib increased and eCF506 decreased phosphorylation of this residue in a concentration-dependent manner in both cell lines. FAK-Y397 is autophosphorylated in response to integrin and growth factor signaling, which creates a docking site for SRC to bind via its SH2 domain, which in turn phosphorylates multiple sites on FAK (Y576/577, Y861, Y925). To rule out the direct inhibition of FAK kinase by eCF506 (note that, as shown in Supplementary Table S3, eCF506 does not inhibit FAK at 1 μmol/L), we studied the effect of the inhibitor on FAK-pY397 in SYF cells, murine embryonic fibroblasts that are deficient for SRC, YES, and FYN. Western blot analysis of lysates from SYF cells treated with either eCF506 or dasatinib showed no concentration-dependent changes in FAK-pY397 levels (Supplementary Fig. S8A, Supplementary Materials and Methods), further evidence that these inhibitors do not directly affect FAK kinase activity. Western blot analysis of the negative regulatory site of SRC-pY530 after treatment with eCF506 showed that the reduction of FAK-pY397 levels correlated with a minor increase in the phosphorylation of SRC-Y530, whereas the opposite results were observed with dasatinib (Supplementary Fig. S8B, Supplementary Materials and Methods). These data suggest there is association between the SRC structure and FAK-Y397 phosphorylation status.

### Dasatinib, but not eCF506, triggers FAK translocation into the nucleus

Coimmunoprecipitation and Western blot assays demonstrated that SRC inhibition mediated by dasatinib or eCF506 induces opposite effects on SRC–FAK complex formation and FAK-pY397. The question remains whether these contrasting activities lead to different effects on FAK function. Although FAK's main role is to act as a scaffold for focal adhesions at the cell membrane, FAK is also found in the nucleus under cellular stress (43, 44), where it acts as a transcriptional modulator. The effects of dasatinib and eCF506 on FAK localization were then investigated by subcellular fractionation in MDA-MB-231 cells (Supplementary Fig. S8C, Supplementary Materials and Methods). Western blot analysis shows that SRC kinase predominantly localizes to the perinuclear fraction, whereas FAK is found in the cytoplasmic and perinuclear fractions. Notably, dasatinib but not eCF506 increases the amount of nuclear FAK, indicating that FAK shuttling from the cytoplasm to the nucleus is triggered by dasatinib. These results were corroborated in squamous cell carcinoma (SCC) cells (Supplementary Fig. S9A, Supplementary Materials and Methods). Coimmunoprecipitation studies of the nuclear fraction confirmed that FAK is bound to SRC after dasatinib treatment (Supplementary Fig. S9B, Supplementary Materials and Methods). The contrasting effects mediated by dasatinib and eCF506 on the nuclear translocation of FAK are important because the presence of FAK in the nucleus is associated with gene expression changes that promote tumor evasion of immune surveillance (45).

We then investigated a murine breast cancer cell model to produce syngeneic grafts in mice with an intact immune system. MetBo2 cells are murine triple negative-like breast cancer cells derived from bone homing cells following intracardiac inoculation of murine Met1 cells (46, 47) and genetically modified to express firefly luciferase. Western blot analysis of the MetBo2 cells (Supplementary Fig. S10A, Supplementary Materials and Methods) confirmed high levels of Src-pY418 (mouse equivalent to human SRC-pY419). Cell viability assays and immunoblotting showed that both dasatinib and eCF506 elicit potent inhibition of MetBo2 proliferation (Supplementary Fig. S10B, Supplementary Materials and Methods), whereas eCF506 induces more potent Src-pY418 inhibition than dasatinib at the same concentrations (Supplementary Fig. S10C, Supplementary Materials and Methods). Subcellular fractionation studies confirmed that dasatinib also triggers the translocation of FAK into the nucleus in this cell line, but not eCF506 (Supplementary Fig. S9, Supplementary Materials and Methods).

### eCF506 shows excellent tolerability and potent SRC inhibition *in vivo*

Toxicology studies were performed to inform on eCF506 safety and experimental design before *in vivo* testing. Along with earlier studies that established eCF506 is a weak inhibitor of CYP enzymes and hERG channel activity (36), a negative Ames test confirmed that eCF506 is not mutagenic (Supplementary Fig. S11, Supplementary Materials and Methods). Single-dose acute toxicity studies in mice and rats determined that eCF506's MTD by oral administration was greater than 200 mg/kg in both species, regardless of the gender (see full results in Supplementary Tables S6 and S7, Supplementary Figs. S12A and S12B and S13A and S13B, Supplementary Materials and Methods), and >400 mg/kg in female mice.

We then investigated eCF506 exposure after a single oral dose in female CD1 nude mice (study performed by Evotec). Three mice per group were dosed with 40 or 80 mg/kg of eCF506 and analyzed at 2, 8, and 24 hours. Dose proportionality was observed for eCF506's blood levels between the doses (Supplementary Figs. S14A and S14B, Supplementary Materials and Methods). A single oral dose of 40 mg/kg achieved >0.2 μmol/L concentration of eCF506 in blood for 24 hours, levels that in accordance with the MetBo2 cell studies (Supplementary Fig. S10, Supplementary Materials and Methods) would be sufficient to fully inhibit Src activity.

**Figure 4.**
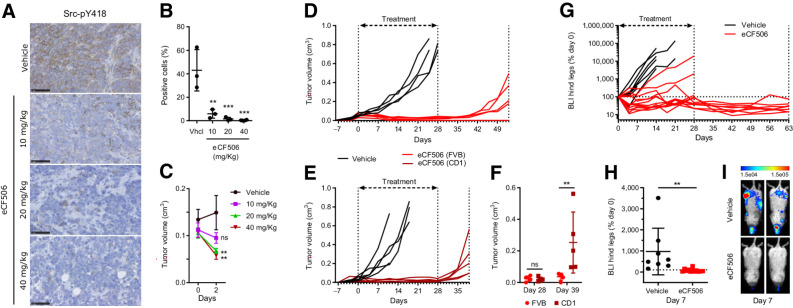
**A–C,** *In vivo* PD study of Src-pY418 inhibition. CD1 nude female mice implanted with MetBo2 cells (left fourth mammary fat pad; approximate tumor volume = 0.1 cm^3^) were randomized in four groups and treated daily for 3 days (oral) with eCF506 at 10, 20, and 40 mg/kg or vehicle. **A,** Representative IHC images of tumor sections immunostained using anti-Src-pY418 antibody (brown) and counterstained with hematoxylin (blue). **B,** Quantitative analysis of Src-pY418 positive cells from the IHC images. Error bars, ±SD from *n* = 3. **, *P* < 0.01; ***, *P* < 0.005 (ANOVA). **C,** Comparison of tumor size between groups at day 0 and 2. Error bars, ±SD from *n* = 3. **, *P* < 0.01 (ANOVA). **D** and **E,***In vivo* study of breast tumor growth over time in immunocompetent FVB (**D**) and immunocompromised CD-1 nude (**E**) mice during and posttreatment with vehicle or eCF506. Female mice implanted with MetBo2 cells (left fourth mammary fat pad; approximate tumor volume = 0.04–0.05 cm^3^) were randomized in two groups and treated daily for 28 days (oral) with eCF506 (40 mg/kg) or vehicle. After treatment completion, animals were monitored until tumor relapse. **F,** Comparative analysis of tumor volumes at days 28 and 39 in FVB wild-type vs. CD1 nude mice treated with eCF506. Error bars, ± SD from *n* = 5/group. **, *P* < 0.01 (*t* test). **G–I,***In vivo* study of metastasis inhibition by eCF506 in syngeneic murine breast cancer bone metastasis model. Female FVB mice injected with MetBo2 cells (left ventricle) were randomized in two groups 7 days after the injections and treated daily for 28 days (oral) with eCF506 (40 mg/kg) or vehicle. **G,** Percentage BLI in left and right hind legs (two signals per animal) of vehicle- and eCF506-treated mice relative to day 0. Animals were monitored for 63 days. **H,** Comparative analysis of % BLI between groups at day 7. Error bars, ± SD from *n* = 8–10/group. **, *P* < 0.01 (*t* test). **I,** Bioluminescence tomography images of two representative mice from each group at day 7.

Next, we studied the optimal dose range required to achieve effective inhibition of Src-pY418 in an orthotopic murine breast cancer model. MetBo2 cells were injected into the mammary fat pad of female mice (CD1 nude strain) and tumors allowed to grow up to approximately 0.1 cm^3^. Mice were dosed daily for 3 days with eCF506 (10, 20, or 40 mg/kg, in 3 mmol/L citrate buffer) or vehicle (3 mmol/L citrate buffer) by oral gavage and culled 3 hours after the last dose (*n* = 3/group). Tumors were fixed, sections labeled for Src-pY418 and stained with hematoxylin. As shown in Fig. 4A and B, IHC analysis demonstrated significant reduction of Src-pY418 in the allograft sections from mice treated with eCF506 at all doses relative to the vehicle control, with the 40 mg/kg dose achieving near complete inhibition. Of note, target inhibition correlated in a dose-dependent manner with a reduction of tumor volume at day 2 of the treatment (Fig. 4C).

### eCF506 shows enhanced antitumor activity in immunocompetent cancer models

Next, we tested the *in vivo* antitumor activity of eCF506 in primary breast cancer models in immunocompetent (FVB wild type) and immunocompromised (CD1 nude) mice. MetBo2 cells were injected into the mammary fat pad of female mice and, after 8 days, treated them daily with vehicle (3 mmol/L citrate buffer) or 40 mg/kg eCF506 by oral gavage. Animals' weight remained stable and comparable between the treatment groups during the length of the study (Supplementary Figs. S15A and S15B, Supplementary Materials and Methods). Administration of eCF506 was stopped after 28 days and tumor growth monitored in the absence of treatment until tumor relapse. As shown in Fig. 4D and E, potent antitumor activity was observed in all the mice treated with eCF506 from both immunocompetent (Fig. 4D) and immunocompromised (Fig. 4E) strains during the treatment phase, confirming the *in vivo* efficacy of eCF506 and the sensitivity of MetBo2 allografts to Src inhibition. Notably, major differences were observed during the posttreatment phase. Residual tumors regrew rapidly in CD1 nude mice after cessation of eCF506 treatment, undergoing >5-fold volume increment from days 28 to 39 (Fig. 4F). In contrast, tumor relapse was delayed by more than 11 days in immunocompetent mice following treatment cessation (Fig. 4D and F) and one mouse remained tumor-free until the end of the study (53 days), suggesting involvement of the immune system in the control of tumor growth during the posttreatment phase.

We next tested eCF506 in a bone metastasis model established by intracardiac injection of luciferase-expressing MetBo2 cells into the left ventricle of FVB mice, and monitored by bioluminescence tomography (Optima imager, Biospace). It is reported that metastatic breast cancer cells that lodge in the bone marrow become addicted to SRC signaling (47), what would make them highly susceptible to eCF506 treatment. The performance of this metastasis assay was also inspired by the previously reported capacity of eCF506 to inhibit cell migration (36). Metastatic lesions were allowed to grow for 7 days until the luminescence signals were clearly detected. Mice mainly developed tumors in the hind legs (also in the jaw bones) and were randomized into two treatment groups: vehicle (4 animals) or 40 mg/kg eCF506 (5 animals). The bioluminescence intensity (BLI) of tumors of each hind leg was analyzed twice a week and plotted in Fig. 4G. Animals were culled when the tumor burden became too great, which was achieved for the vehicle group at days 7 to 21. Strong anticancer effect was observed in all mice treated with eCF506 (Fig. 4H and I), inducing regression of hind leg metastases in 4/5 mice during the treatment phase. As in previous studies, monitoring of surviving mice (4) was continued after the treatment period. The residual BLI in the hind legs of these mice remained below detectable levels during the following 7 weeks (Fig. 4G). Although 2/4 mice developed metastatic lesions in the thoracic cavity before the study termination (see imaging analysis in Supplementary Figs. S16A and S16B, Supplementary Materials and Methods), no bioluminescence signal was observed from hind legs or bone jaw during the posttreatment phase, which indicates that bone metastases were completely suppressed by the 28-day eCF506 treatment in 4 of 5 mice treated.

**Figure 5.**
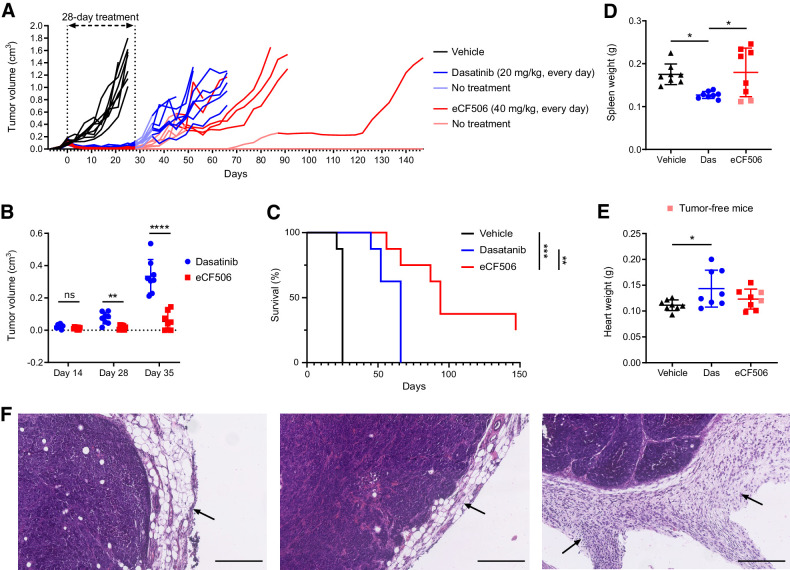
**A,** *In vivo* study of tumor growth inhibition by eCF506 and dasatinib in immunocompetent mice. Female FVB mice implanted with MetBo2 cells (left fourth mammary fat pad; approximate tumor volume = 0.1 cm^3^) were randomized in three groups and treated daily for 28 days (oral) with dasatinib (20 mg/kg; blue), eCF506 (40 mg/kg; red), or vehicle (black). Tumors were measured twice per week. After day 28, animals were monitored until tumor relapse and a second treatment phase initiated when tumors reached a volume of 0.3 cm^3^. **B,** Comparative analysis of tumor volumes of dasatinib- and eCF506-treated arms at day 14, 28, and 35. Error bars, ± SD from *n* = 8/group. **, *P* < 0.01; ****, *P* < 0.001 (*t* test). **C,** Kaplan–Meier survival plot showing deaths due to tumor size or sickness for each treatment group. Statistical analysis by log-rank (Mantel–Cox) test. **D** and **E,** Comparative analysis of spleen (**D**) and heart weight (**E**) for vehicle-, dasatinib-, and eCF506-treated groups at mouse endpoint. Error bars, ±SD from *n* = 8/group. *, *P* < 0.05 (ANOVA). Note that cured eCF506-treated mice (light red points), which did not need to react to the tumor threat for 120 days, featured smaller spleens than the rest of the group. **F,** H&E-stained histology images of tumors after treatment with vehicle (left), dasatinib (middle), and eCF506 (right). Black arrows point to the peritumoral chronic inflammatory infiltrates in surrounding adipose tissue to highlight the differences between the groups. Scale bar, 250 μm.

Finally, we performed a head-to-head efficacy study between dasatinib and eCF506 in the primary breast cancer model in immunocompetent FVB mice (*n* = 8/group). For consistency, the treatment phase was run for 28 days. Dasatinib and eCF506 were dosed daily by oral gavage at 20 and 40 mg/kg, respectively. Dasatinib's dose of 20 mg/kg was chosen to achieve maximal therapeutic effect and good tolerability, since higher repetitive doses can result in severe toxicity and lethality (48–50). The weight of the animals was monitored during the length of the study, which remained comparable between groups (Supplementary Fig. S17, Supplementary Materials and Methods). Potent antitumor response was mediated by both inhibitors during the treatment phase (Fig. 5A), displaying equivalent anticancer effect at day 14 (Fig. 5B). However, whereas tumor growth was halted in the eCF506-treated arm throughout the 28 days treatment period, a significant increment in tumor volume was observed in the dasatinib-treated group by the end of the treatment period (day 28, see Fig. 5B). Notably, the posttreatment phase revealed increasing differences between the treatment groups, with rapid tumor relapses occurring in the absence of treatment in the dasatinib-treated animals. In contrast, tumor growth was significantly more delayed in the mice treated with eCF506 (Fig. 5B). To test the response of the relapsed MetBo2 allografts to inhibitors' retreatment, we initiated a second phase of treatment in those animals whose tumors reached an approximate size of 0.3 cm^3^. The retreatment phase started at day 35 for the dasatinib-treated group. Following drug dosing, tumors underwent partial regression, but started to regrow after a small number of repeated dosing. The tumors of all the mice treated with dasatinib evolved similarly throughout the different phases of the study and had to be sacrificed at days 44 to 66. In contrast, the tumors of the eCF506-treated group relapsed at different growth rates. Consequently, the second phase of treatment was initiated on different dates for each mouse: on day 39 (*n* = 2), 43 (*n* = 3), and 87 (*n* = 1). Notably, 2 of 8 eCF506-treated mice remained tumor-free until the study endpoint (148 days) without the need of a second treatment phase. Overall, the antitumor effect mediated by eCF506 in immunocompetent mice was superior to that of dasatinib, resulting in highly significant differences in survival (Fig. 5C). To investigate potential reasons behind these variances, spleens, hearts, and tumors were analyzed post-mortem (see Fig. 5D–F). The size of the spleens was significantly smaller in the dasatinib-treated arm compared with the vehicle and eCF506-treated groups (Fig. 5D). Histologic analysis did not find differences between the spleens of any of the groups (Supplementary Fig. S18, Supplementary Materials and Methods), suggesting that the reduced spleens found in the dasatinib-treated arm are not a sign of splenotoxicity but a consequence of decreased spleen function, in agreement with toxicology studies performed in other animal models (50). Analysis of tumor sections identified important differences between the groups in peritumoral chronic inflammation and lymphocyte infiltration at the surrounding adipose-rich connective tissue (Fig. 5F; Supplementary Fig. S19, Supplementary Materials and Methods). Moderate to marked lymphocyte infiltration was seen in eCF506-treated tumors, but only mild for the vehicle control and dasatinib-treated tumors. IHC staining of tumor sections for CD3^+^ cells indicated that eCF506 treatment leads to increased levels of tumor infiltrating T cells compared with vehicle and dasatinib treatment groups (Supplementary Fig. S20). However, large necrotic areas (light brown) were also seen in the tumor samples treated by eCF506, which may be in part responsible for the elevated lymphocyte infiltration. As seen in Fig. 5E, the hearts of the dasatinib-treated arm were significantly heavier compared with the control or eCF506 treatment. H&E analysis showed focal myocyte hydropic change and variation in myocyte cell size only in animals treated with dasatinib (Supplementary Figs. S21A and S21B, Supplementary Materials and Methods). This is one of the expected liabilities of ABL inhibition that limits dasatinib's therapeutic index (32–34, 50).

Because of the difficulty to compare immune cell changes from long-term treated samples, we next performed a short-term study in the immunocompetent orthotopic MetBo2 breast cancer model (*n* = 8 mice/group) by treating animals orally for 5 days with vehicle, dasatinib (20 mg/kg) or eCF506 (40 mg/kg). Mice were culled at endpoint and the weights of tumors, spleens, and hearts measured (see Supplementary Fig. S22A, Supplementary Materials and Methods). In agreement with prior studies, tumors were significantly reduced in the animals treated with dasatinib or eCF506. Notably, although hearts were unaffected, the weights of the spleens were significantly reduced in the dasatinib-treated group relative to vehicle- and eCF506-treated animals, indicating that the immunosuppressive effects of dasatinib arise shortly after initiating the treatment. Intriguingly, immunophenotyping of tumor samples (*n* = 5/group) by flow cytometry found no differences in T-, B-, and NK-cell abundance between the treatment groups (Supplementary Figs. S22B and S22C, Supplementary Materials and Methods), suggesting that lymphocytes are not directly affected by the treatments, at least after five daily doses. Transcriptomic analysis was then performed with the remaining tumor samples using the PanCancer Immune Profiling panel in the NanoString nCounter platform. As shown in Supplementary Fig. S23, expression levels of nine genes associated with the M1 macrophage phenotype, including CD80, were found differentially regulated in eCF506-treated tumors relative to vehicle- and dasatinib-treated animals. The results from the short-term study suggests that, rather than directly affecting lymphocytic cell population, eCF506 treatment induces changes in the infiltration number and/or reprogramming of myeloid cells. Of note, four M2 associated genes and two biomarkers of tumor-associated macrophages were also differentially regulated in the eCF506-treated arm.

## Discussion

The inherent promiscuity of kinase inhibitors is a double-edged sword that critically influences the therapeutic scope of this class of drugs. Although selected polypharmacologic properties can improve cancer treatment by, for example, blocking redundant signaling pathways and/or mediating additive/synergistic effects, pan-kinase inhibition frequently results in dose-limiting adverse effects and narrow therapeutic indexes (51). Moreover, off-target activities can potentially counteract the on-target therapeutic benefit of the drug, making kinase selectivity a desirable feature not only to promote clinical success, but also to pharmacologically elucidate cause–effect relationships of nominated targets. The kinase inhibitors approved or in clinical development that target SRC activity are classic examples of promiscuous inhibitors, targeting many kinases beyond SRC and its most closely related family members. In fact, they all are dual SRC/ABL inhibitors with additional off-target activities dependent on the inhibitor. Here we show that eCF506 inhibits SRC more potently than any other kinase and displays low to no inhibition of 315 kinases of 340 tested (including ABL). Remarkably, co-crystal structure analysis reveals that eCF506 induces and stabilizes the native inactive conformation of the SRC kinase domain, where the C-helix is displaced away from the active site and breaks the salt bridge between E313 in the C-helix and the catalytic K298 (39, 52). The limited availability of SRC inhibitors displaying this mode of binding combined with the benefits of targeting inactive kinase conformations make this both a remarkable and meaningful finding that allows us to interpret the properties of eCF506. Binding to inactive forms of kinases often increase selectivity resulting from highly divergent inactive kinase structures. Imatinib, for example, targets the inactive conformation of the ABL kinase and shows high selectivity over SRC. This is because imatinib targets an inactive ABL conformation where the Asp side chain of the catalytic DFG motif flips from pointing into the active site (DFG-*in*) to point away from the active site (DFG-*out*), thereby switching its kinase activity off (53), a feature rarely seen in SRC family kinases. In contrast, ABL is not thought to natively use the C-helix-*out* conformation as an “off-switch.” Hence the mode of binding of eCF506 is optimal for inhibiting SRC but not ABL, providing a sound explanation for this unique selectivity feature. Structurally speaking, the mode of binding of eCF506 to SRC (DFG-*in*, C-helix-*out*) is analogous to that of the dual HER2/EGFR inhibitor lapatinib on its targets (54). Lapatinib does not inhibit SRC family members, while eCF506 does not inhibit HER2 or EGFR (see Supplementary Table S1, Supplementary Materials and Methods), which illustrates the selectivity offered by this binding mode.

A second advantage of targeting inactive conformations of kinases is that it can affect noncatalytic scaffolding functions, in that inactive kinases are often excluded from functional complexes with binding partners. We show that the inactive conformation of the SRC kinase domain induced by eCF506 is relayed to the full-length protein, keeping the protein in a compact conformation, likely as seen in the autoinhibited SRC structure (55). In cells, we show that eCF506 reduces SRC binding to its partner FAK. Notably, we also found that the SRC/ABL inhibitor dasatinib (known to bind SRC in its active conformation; ref. 53) induces precisely the opposite effect. These results provide convincing evidence that eCF506 and dasatinib bind and promote different conformations in SRC and endorse the re-classification of eCF506 into a new functional category: “total SRC inhibitor,” which inhibits both SRC kinase activity and scaffolding functions.

Screening across a panel of 16 breast cancer cell lines shows that the on-target potency and selectivity of eCF506 translate into high antiproliferative effects against triple negative and ER+ breast cancer cells, by inhibition of the cell cycle. We also show that eCF506 inhibits SRC kinase activity with greater potency than any other SRC/ABL inhibitor in all the cell lines tested, requiring 0.03 to 0.1 μmol/L to achieve full inhibition. Importantly, the antiproliferative potency of eCF506 in the sensitive breast cancer cells correlated well with the observed inhibition of SRC-pY419, although the potential contribution of partly inhibiting other targets (e.g., BRK) cannot be ruled out. In agreement with the literature (41), our results indicate that SRC activity has a minor role on the proliferation and survival of HER2-driven breast cancer cell lines, but it can become a driver of resistance in the context of HER2 inhibition.

A remarkable observation that further differentiates eCF506 from other SRC inhibitors is its indirect effect on the autophosphorylation site of FAK, Y397. Mirroring the effect mediated by FAK inhibitors, eCF506 reduces the levels of FAK-pY397 relative to the untreated control. In contrast, dasatinib treatment results in a marked increment of FAK-Y397 phosphorylation. The changes induced on FAK-pY397 by each inhibitor inversely correlates with the effect on SRC-pY530, the negative regulatory site of SRC. Given that neither eCF506 nor dasatinib directly affect FAK activity, we propose that the driving force behind the changes on FAK-pY397 are the opposing effects mediated by these inhibitors on the formation of the SRC–FAK complex (Fig. 2A and C). Phosphorylation and dephosphorylation of FAK is a dynamic process required for the assembly and disassembly of focal adhesions. When SRC is forced into a closed conformation by eCF506, its SH2 domain is not available to bind to the FAK-pY397 docking site and form the active complex with FAK. This in turn overexposes the pY397 site of FAK to enzymatic dephosphorylation (56). In contrast, dasatinib's promotion of SRC–FAK binding protects this site from dephosphorylation.

In agreement with our findings, Higuchi and colleagues have recently reported that type I SRC inhibitors like dasatinib are allosteric facilitators of SRC conformational activation, enhancing SRC–FAK complex formation and increasing FAK autophosphorylation (57). They also observed that a decrease of dasatinib concentration leads to activation of SRC–FAK signaling and cautioned about the possibility that type I SRC kinase inhibitors can evoke growth signals *in vivo* when the drug concentration decreases, thus accelerating cancer growth. The alternative mode of inhibiting SRC mediated by eCF506 would not lead to such paradoxical activation, which may be a critical advantage to its clinical use.

Another result that highlights the distinct molecular consequences of targeting different conformations of SRC is the effect in the translocation of FAK into the nucleus. We show that dasatinib triggers the translocation of FAK into the nucleus and that nuclear FAK is found in complex with SRC. On the contrary, SRC but not FAK can be detected in the nuclear fraction of cells treated with eCF506, indicating that the active conformation of SRC is required to traffic FAK into the nucleus.

We show that eCF506 mediates very potent *in vivo* antitumor activity in murine triple-negative breast cancer models, against primary tumors and bone metastases, regardless of the mouse strain used. Nonetheless, we also show in the primary breast cancer model that significant differences emerge in the posttreatment period: mice with an intact immune system are capable to control tumor growth beyond the treatment phase (> 10 days), whereas tumor relapse occurs in immunosuppressed mice as soon as the animals are off treatment. Emulating the results of eCF506 in immunosuppressed mice, we show that dasatinib elicits a strong anticancer effect in immunocompetent mice during the treatment phase, but tumor growth accelerates immediately after halting the treatment. The posttreatment antitumor effect observed in the eCF506-treated arm (25% of mice remained tumor-free after the initial treatment phase) along with the higher response of most mice to the second treatment phase, resulted in significantly superior survival of the animals treated with eCF506 relative to dasatinib. Post-mortem analysis of the animals revealed smaller spleens in the dasatinib-treated group than in those treated with vehicle or eCF506. Notably, this effect was also observed in the short-term study (five daily doses). Taking into consideration that normal splenic histology was observed in all the treatment groups, dasatinib's effects are consistent with spleen hypoactivity (50). This is relevant because a reduction of tumor-fighting effector cells can affect the capacity of the adaptive immune system to respond to the cancer threat (58, 59). This is also supported by the histologic analysis of the tumors, which revealed a higher presence of peritumoral lymphocytes and CD3^+^ cells in the eCF506-treated mice compared with the dasatinib group and the vehicle control. However, the results from the short-term study suggests that macrophage reprogramming may be involved in the initiation of the differentiating phenotypic effects observed by eCF506 treatment. The overall pro-immune effects of eCF506 may be associated with its high selectivity over kinases that are targeted by dasatinib, such as ABL or KIT (see Supplementary Table S3). Although dasatinib has shown good tolerability in cancer patients (34), its broad immunosuppressive effects [which have been found to benefit CML patients by reducing regulatory T cells (60), but it also results in the suppression of cytotoxic T-cell function (50, 61) and myelosuppression (62)] is a potential disadvantage to treat solid malignancies in immunocompetent animals. Finally, we show that some of the animals of the dasatinib-treated group presented significant enlargement of the heart and damaged cardiac muscle tissue. This adverse effect, which is attributed to Abl inhibition (33, 34, 50), was not observed in the eCF506-treated group, which agrees with the superior safety profile of this selective inhibitor.

In conclusion, we report here a mode of inhibiting SRC that impairs both catalytic and scaffolding functions by locking the protein in its native inactive conformation. Molecular and phenotypic data in breast cancer models, both *in vitro* and *in vivo*, demonstrate that this inhibition mode confers therapeutic advantages compared with classical type I inhibitors. Due to its selectivity and mechanism-of-action, we anticipate that eCF506 will become a tool of choice to assist in the elucidation and characterization of the role of SRC function in different pathologies. Finally, based on its *in vivo* potency and tolerability, we support the nomination of this total SRC inhibitor as a first-in-class clinical candidate for the treatment of SRC-associated disorders.

## Authors' Disclosures

C. Fraser reports grants from Wellcome Trust during the conduct of the study; also has a patent for EP3298015BA issued, a patent for JP6684831B2 issued, a patent for US10294227B2 issued, a patent for CN107849050B issued, and a patent for CA3021550A1 pending. B-Z. Qian reports personal fees and other support from MedAnnex Limited outside the submitted work. N.O. Carragher reports grants from Wellcome Trust during the conduct of the study; also has a patent for EP3298015B1 issued, a patent for JP6684831B2 issued, a patent for US10294227B2 issued, a patent for CN107849050B issued, and a patent for CA3021550A1 pending. A. Unciti-Broceta reports grants from Wellcome Trust during the conduct of the study; also has a patent for EP3298015B1 issued, a patent for JP6684831B2 issued, a patent for US10294227B2 issued, a patent for CN107849050B issued, and a patent for CA3021550A1 pending. No disclosures were reported by the other authors.

## Supplementary Material

Supplementary Data

Supplementary Data

Supplementary Data
