# Supplementary material for: A Conformation Selective Mode of Inhibiting SRC Improves Drug Efficacy and Tolerability
Source: Cancer Res. 2021 Aug 20;81(21):5438–50. doi: 10.1158/0008-5472.CAN-21-0613 (PMC7611940; doi:10.1158/0008-5472.CAN-21-0613)
Supplement: Supplementary Data [file can-21-0613_supp.pdf]

METHODS

Crystal structure of SRC bound to eCF506

Expression and purification. The hSrc kinase domain (residues 254-536) or hSrc-SH3-SH3-kinase (84-536) were expressed with an N-terminal 6xHis tag in E.coli (BL21) co-expressed with the YopH phosphatase, as described in ref. *1*. Pure protein was obtained by Ni-chelate affinity chromatography (HisTrap, GE Healthcare), ion exchange chromatography (Source 15Q column, GE Healthcare) and size exclusion chromatography (Superdex200, GE Healthcare). After the Ni-chelating column, the 6xHis tag was removed by incubation with TEV protease.

Crystallization. Purified hSrc kinase was concentrated to 6.3 mg/ml (193 µM) and incubated with 10 mM eCF506 for 30 min on ice. Crystallisation was performed using the hanging drop, vapor diffusion method. Crystallization conditions were optimized from ref. *2* with final crystals grown by mixing the protein with equal volume of 6% PEG3350, 300 mM ammonium acetate, 0.1M HEPES pH 7.5, 10 mM TCEP. Crystals were flash frozen in liquid nitrogen after soaking in growth condition with 25% ethylene glycol.

Data collection and structure solution. Diffraction data collection was carried out at the XALOC beamline of the ALBA synchrotron facility (Barcelona, Spain). Data was integrated to 1.5 Å using XDS (*3*), scaled with AIMLESS (*4*) and an initial structure obtained by molecular replacement with PHASER (*5*) using the Src kinase domain (PDB: 4MXO) as search probe. The structure was refined using Refmac5 (*6*) and manual rebuilding with Coot (*7*). Final R-factors are 18.0/21.3 (R_{work}/R_{free}). Final crystallographic and refinement statistic are listed in Table S1.

Biophysical characterization of SRC hydrodynamic radius

Sedimentation velocity analytical ultracentrifugation (SV-AUC). SV-AUC assays were performed at the Molecular Interactions Facility at the CIB Margarita Salas. Protein samples at concentrations

of 15 μM in 20 mM Tris-HCl, 150 mM NaCl and 1 mM TCEP pH 8.0 buffer, were loaded (320 μL) into analytical ultracentrifugation cells. SV-AUC assays were carried out at 20 ºC and 48,000 rpm in a XL-I analytical ultracentrifuge (Beckman-Coulter Inc.) equipped with both UV-VIS absorbance and Raleigh interference detection systems, using an An-50Ti rotor, and 12 mm Epon-charcoal standard double-sector centerpieces. Sedimentation profiles were recorded at 290 nm. Differential sedimentation coefficient distributions were calculated by least-squares boundary modelling of sedimentation velocity data using the continuous distribution c(*s*) Lamm equation model as implemented by SEDFIT (*8*). These *s* values were corrected to standard conditions (water, 20 °C, and infinite dilution) (*9*) using the program SEDNTERP (*10*) to get the corresponding standard *s* values ($s_{20,w}$).

Dynamic Light Scattering (DLS). DLS experiments were carried out at the Molecular Interactions Facility at the CIB Margarita Salas. The experiments were performed using a Protein Solutions DynaPro MS/X instrument (Protein Solutions) at 20 °C and a 90° light scattering cuvette. The samples were centrifuged during 10 min at 12,000 g and 20 °C immediately before measurements. Data were collected and analysed with the Dynamics V6 Software.

Kinome profiling of eCF506

Screenings were performed by Reaction Biology Corp. using a radioisotope-based assay ([γ-^{33}P] ATP) consisting of measuring ^{33}P incorporation on the substrate (poly [Glu, Tyr] 4:1) relative to DMSO. For the whole kinome screen eCF506 was screened against 340 wild type kinases at a single dose of 1 μM, in duplicate, with 10 μM of ATP. Enzymatic activity values relative to DMSO (considered as 100% of activity) were averaged and plotted in Table S3 (see comparison with dasatinib and bosutinib in Table S4).

Thermal shift assays

The protocol was adapted from Jafari *et al.* (*11*). Cells were seeded (3 million / 10 cm dish) and left to attach for 24 h. Compounds were added (final DMSO concentration 0.1% v/v) and cells incubated for 1 h before being washed with PBS, trypsinized, collected in PBS and centrifuged (300 g, 3 min). The cell pellet was gently resuspended in 10 mL PBS, centrifuged again (300 g, 3 min), resuspended in 1 mL PBS with protease inhibitors (aprotinin, leupeptin) and compounds were added back to samples at the previously used concentrations. Each sample was split into 10 PCR tubes (100 µL) which were heated at different temperatures (40.0 °C to 65.0 °C) for 3 min. Samples were cooled at room temperature for 3 min before being put on ice, lysed in 100 µL of a mild detergent lysis buffer prepared at 2X (40 mM Tris HCl pH 7.5, 300 mM NaCl, 1 % v/v Triton X100), briefly vortexed and centrifuged at 4 °C (17,000 g, 20 min). The supernatant was transferred into a new microcentrifuge tube and stored at -20 °C. Standard Western blots were performed and relative protein amounts quantified to assess heat degradation of the compounds' protein targets compared to DMSO controls.

Cell-based screenings

For cell proliferation assays, cells were seeded in 96-well plates as specified below.

Cell line	Medium	Supplements	Cells / well
**BT-474**	RPMI	10% FBS, 2 mM L-glutamine	5,000
**BT-549**	RPMI	10% FBS, 2 mM L-glutamine	1,000
**CAMA-1**	DMEM	10% FBS, 2 mM L-glutamine	1,000
**HCC-1569**	DMEM	10% FBS, 2 mM L-glutamine	3,000
**HCC-1954**	DMEM	10% FBS, 2 mM L-glutamine	2,000
**JIMT-1**	DMEM	10% FBS, 2 mM L-glutamine	1,000
**KPL-4**	DMEM	10% FBS, 2 mM L-glutamine	1,000
**MCF-7**	DMEM	10% FBS, 2 mM L-glutamine	1,000
**MDA-MB-134VI**	RPMI	10% FBS, 2 mM L-glutamine	10,000

**MDA-MB-157**	DMEM	10% FBS, 2 mM L-glutamine	5,000
**MDA-MB-231**	DMEM	10% FBS, 2 mM L-glutamine	1,000
**MDA-MB-436**	DMEM	10% FBS, 2 mM L-glutamine	3,000
**MetBo2**	DMEM	10% FBS, 2 mM L-glutamine	1,000
**SCC**	MEM	10% FBS, 2 mM L-glutamine, 1X MEM non-essential amino acids, 1X MEM vitamins, 1 mM sodium pyruvate; 1% Hygromycin	
**SKBR3**	DMEM	10% FBS, 2 mM L-glutamine	4,000
**SUM44PE**	Ham's F-12	1 g/L BSA, 5 mM ethanolamine, 10 mM HEPES, 1 µg/mL hydrocortisone, 5 µg/mL insulin, 50 nM sodium selenite, 5 µg/mL apo-transferrin, 10 nM triiodo thyronine; 2% FBS was added when thawing and splitting cells.	5,000
**SYF**	DMEM	10% FBS, 2 mM L-glutamine	
**T47-D**	RPMI	10% FBS, 2 mM L-glutamine	2,000
**ZR75.1**	RPMI	10% FBS, 2 mM L-glutamine	4,000

Cell cycle assay

Cells were seeded (1 million cells / 15 cm plate) and left to attach for 24 h. Compounds were added (final DMSO concentration 0.1% v/v) and the treated plates were incubated for 48 h. Cells were trypsinized, resuspended in medium and centrifuged (1,000 rpm, 5 min). The cell pellet was resuspended in 50 µL PBS with 2 % FBS and cell aggregates were broken up through gentle pipetting. Cells were fixed by adding 1 mL ice-cold 70 % ethanol dropwise while vortexing gently and samples were stored at 4 °C for up to two weeks. The cell concentration was determined using a hemocytometer and 1.5 million cells were used per sample. Cells were centrifuged (1,000 rpm, 5 minutes) and the cell pellet was resuspended in 2 mL pre-warmed pepsin solution and incubated for 30 min in a 37°C water bath with frequent vortexing. The digested samples were centrifuged (4,000 rpm, 4 minutes) and the pellet was resuspended in 0.25 mL HCl and incubated for 15 min at room temperature. 12 mL PBS was added and samples were centrifuged (4,000 rpm, 4 minutes), after which the pellet was washed a second time in 5 mL PBS. The pellet was resuspended in 0.5

mL DAPI solution and incubated in the dark for 30 min at room temperature. Samples were analyzed by flow cytometry using the LSR Fortessa (BD Biosciences) and 30,000 events were recorded per sample.

Western blotting

List of antibodies used for western blot assays.

Antibody list. Antibodies for Western blots were diluted in blocking buffer with 0.02% w/v sodium azide.

Target	Host	Company	ID	Dilution
**Western blots**				
α-tubulin	Mouse	CST	3873	1 / 1000
β-actin	Mouse	CST	3700	1 / 1000
β-catenin	Rabbit	CST	9582	1 / 1000
BRCA1	Rabbit	CST	9010	1 / 1000
FAK	Rabbit	CST	3285	1 / 1000
FAK (clone 4.47)-agarose	Mouse	Millipore	05-537	5 μg
FAK-pY397	Rabbit	CST	3283	1 / 1000
FAK-pY861	Rabbit	Invitrogen	44626	1 / 1000
GAPDH	Rabbit	CST	5174	1 / 1000
GM130	Mouse	BD	610822	1 / 1000
Histone H4	Mouse	CST	2935	1 / 1000
mTOR (S2448)	Rabbit	CST	2971	1 / 1000
Rb	Mouse	CST	9309	1 / 1000
SRC	Rabbit	CST	2109	1 / 1000
SRC	Mouse	SCB	SC-8056	1 / 1000
SRC-pY416 (419)	Rabbit	CST	2101	1 / 1000
SRC-pY527 (530)	Rabbit	CST	2105	1 / 1000
Anti-Mouse	Horse	CST	7076	1 / 5000
Anti-Rabbit	Goat	CST	7074	1 / 5000

Proteomics study

The general procedure for the reverse phase protein array has previously been published by Macleod *et al.* (*13*). Cells were seeded in 6-well plates (MDA-MB-231 400,000 cells / well; MCF-7 600,000 cells / well) and left to attach for 24 h. Compounds were added (final DMSO concentration 0.1% v/v) and plates incubated for 3 or 24 h. Cells were washed twice with 3 mL PBS and lysed with 80 µL CLB1 (Bayer Technology Services), scraped and collected in microcentrifuge tubes and incubated at room temperature for 30 min with occasional vortexing. Lysates were centrifuged (17,000 g, 10 min) and the supernatant transferred to new microcentrifuge tubes. The protein concentration was determined in a Bradford Assay using the Coomassie Plus Protein Assay (Thermo Scientific) according to the manufacturer's instructions and all samples were adjusted to 2 mg/mL. 20 µL of the samples were diluted 1:10 with spotting buffer CSBL1 (Bayer Technology Services) and a dilution series (0.2, 0.15, 0.1, 0.05 mg/mL) was created with CSBL1 and 10 % CLB1. The diluted samples were transferred onto a 384-well plate and printed as single spots onto ZeptoChips using the Nanoplotter 2.1E (GeSim) in a controlled environment (70 % humidity, 14 °C). Fluorescently labelled BSA standards were also printed onto the slides. ZeptoChips were blocked for 1.5 h in blocking buffer BB1 (Bayer Technology Services), washed three times for 2 min in distilled water, dried by centrifugation and washed 3 times with 90 µL CAB1 buffer (Bayer Technology Services). Primary antibodies diluted in CAB1 or CAB2 buffer were added and incubated at room temperature overnight, before ZeptoChips were washed three times with CAB1, and 90 µL of appropriate Alexa Fluor conjugated secondary antibodies were added and incubated for 2.5 h in the dark at room temperature. ZeptoChips were washed three times with CAB1, with the last wash left on, and slides scanned using the ZeptoReader (Zeptosens-Bayer). Exposure times just below saturation of the fluorescent signal were automatically selected for analysis by the ZeptoView 3.1 software and a weighted linear fit through the 4-fold dilution series was used to calculate the relative fluorescence intensity (RFI) of

each sample. Normalization to the reference BSA signal was used to compensate for intra- and inter-chip variation. Proteins with low signal were excluded from the analysis. A total of 76 proteins and post-translational modifications were included in the analysis. The local normalized RFI values were then normalized to DMSO controls for each cell line, relative protein abundance expressed as z-scores plotted as heat maps and hierarchical clustering performed (Euclidian distance, complete linkage) using the free online Morpheus software (Broad Institute).

Subcellular fractionation

Cells were seeded in 10 cm dishes and left to attach for 48 h, after which compounds were added (final DMSO concentration 0.1% v/v) and cells incubated for 6 h. Cells were washed twice with ice-cold PBS and 300 µL (10 cm) Buffer A with detergent (20 mM Tris pH 7.5, 1 mM $MgCl_2$, 1 mM EGTA, 1.25 mM PMSF, 0.1 % v/v aprotinin, 100 µM Na_3VO_4, 500 µM NaF, 0.03 % v/v NP-40) was added before cells were gently scraped, collected in microcentrifuge tubes and rotated at 4 °C for 5 min. Samples were centrifuged at 4 °C (800 g, 4 min) and both the supernatant and pellet were kept. The supernatant was transferred into a new tube to which NP-40 was added to a final concentration of 1% v/v, samples vortexed rigorously and centrifuged at 4 °C (17,000 g, 10 min). The supernatant was transferred into a new microcentrifuge tube and labelled as 'cytoplasmic fraction'. The pellet from the previous step was washed once with Buffer A with detergent and once with Buffer A without NP-40 detergent by flicking the tubes until the pellet was suspended, centrifuging at 4 °C (800 g, 3 min) and removing the supernatant. The pellet was resuspended in two volumes of Buffer B (10 mM Tris-HCl pH 7.4, 2.5 mM $MgCl_2$, 1.5 mM KCl, 0.2 M LiCl, 1.25 mM PMSF, 0.1% v/v aprotinin, 100 µM Na_3VO_4, 500 µM NaF, 0.1 % v/v Triton X100, 0.1 % w/v sodium deoxycholate), samples rotated at 4 °C for 15 min, centrifuged at 4 °C (2,000 g, 5 min) and both the supernatant and pellet were kept. The supernatant was transferred into a new tube and centrifuged at 4 °C (17,000 g, 10 min). The supernatant was transferred into a

new microcentrifuge tube and labeled as 'perinuclear fraction'. The pellet from the previous step was washed twice with Buffer B by flicking the tubes until the pellet was suspended, centrifuging at 4 °C (2,000 g, 3 min) and removing the supernatant. The pellet was resuspended in two volumes of RIPA lysis buffer (related to pellet size), sonicated for five cycles (30 s on, 30 s off) and centrifuged at 4 °C (17,000 g, 10 min). The supernatant was transferred into a new microcentrifuge tube and labelled as 'nuclear fraction'. Western blots were performed as described above.

Ames test

The mutagenic potential of eCF506 was assessed by Sequani Ltd (UK) in 4 strains of *Salmonella typhimurium* (TA1535, TA1537, TA98, TA100) and 1 strain of *Escherichia coli* (WP2 uvrA), in the presence and absence of a metabolic activation system (S-9 mix) to allow detection of both point and frame shift mutations. A range of six concentrations of eCF506 up to 5,000 μg / plate were tested in triplicate alongside negative and positive controls (see box below).

The following components were mixed in an Eppendorf tube:

- 2 mL of L-histidine: D-biotin solution (Salmonella) or tryptophan (Escherichia) supplemented top agar.
- 0.1 mL bacterial culture.
- 0.5 mL of S-9 mix *or* 0.2 M phosphate buffer.
- 0.1 mL solvent (negative control) *or* eCF506 dilution *or* positive control.

The mixtures were added to Petri dishes containing minimal agar, which were inverted when set and incubated at 37 ± 3 °C. The integrity of the background lawns was assessed and revertant colonies were scored after approx. 66 ± 1 h using an image analyser. Statistical analysis was carried out using Dunnett's t-test.

Ames test positive controls.

Strain	Without S-9 mix	With S-9 mix
Salmonella typhimurium		
**TA1535**	Sodium azide (1 μg / plate)	2-aminoanthracene (2 μg / plate)
**TA1537**	9-aminoacridine (50 μg / plate)	2-aminoanthracene (4 μg / plate)
**TA98**	2-nitrofluorence (1 μg / plate)	2-aminoanthracene (2 μg / plate)
**TA100**	Sodium azide (1 μg / plate)	2-aminoanthracene (4 μg / plate)
Escherichia coli		
**WP2 uvrA**	4-nitroquinoline-N-oxide (1 μg / plate)	2-aminoanthracene (10 μg / plate)

Animal experiments

Animal experiments were performed under Home Office License in compliance with the Animals (Scientific Procedures) Act 1986 and approved by the University of Edinburgh Ethics Committee.

Acute toxicity

Acute toxicity studies were performed by Evotec (France) to determine the maximum tolerated dose (MTD) of eCF506 following a single oral administration by gavage to male and female Swiss mice and Sprague-Dawley rats. 6 to 8 week-old animals were obtained from Janvier Labs (France) and acclimatized for at least 5 d, and split randomly into groups. eCF506 was formulated in citrate buffer and administered once by oral gavage, with the quantity being adjusted for the respective weight of the animal and received dose being within ± 10% of the theoretical dose. Body weight, temperature and observations for adverse clinical signs or premature death were recorded pre-dosing and 1, 2, 4, 6, 8 and 24 h post-dosing. Blood samples were collected 24 h after dosing from the vena cava and analyzed on a VetScan HM5 hematology analyzer (Abaxis). Post-mortem examinations were performed 24 h after dosing with animals being anesthetized by isoflurane inhalation and exsanguinated prior to cervical dislocation. Necropsy was performed with animals being examined visually for external abnormalities and palpable masses; abnormalities in the

abdominal, thoracic and cranial cavities and contents; and gross abnormalities on organs after their removal.

Immunohistochemistry

Formalin fixed tumours were embedded in paraffin wax blocks using standard procedures by the Pathology Technical Services team (IGMM, University of Edinburgh). Sections (5 µm thick) were cut from paraffin embedded tumours using a microtome, placed on SuperFrost Plus™ slides (Thermo) and dried at 40 °C for 24 h. Sections were dewaxed in xylene, followed by rehydration and antigen retrieval using boiling citrate buffer, pH 6.0, in a pressure cooker (121 °C). Endogenous peroxidases were blocked using Dako REAL Peroxidase-Blocking solution (Agilent, S2023), washed in tap water and were incubated with Dako Serum-free Protein Block (Agilent, X0909), or 2.5% normal goat serum (for B220 rat antibody). Primary antibodies diluted in Dako Background Reducing Antibody Diluent (Agilent, S3022) and incubated with tumour sections at 4 °C overnight. Sections were washed in TBS and incubated with secondary (#P0448 or ImmPRESS HRP Goat Anti-Rat IgG Polymer Detection Kit, Vector Labs, MP-7404-50) for 30 min. Sections were washed in TBS, incubated with DAB reagent (#K3468) for 5 min. Finally, sections were counter-stained with haematoxylin, dehydrated with ethanol and xylene, and mounted using DPX mounting medium (Sigma-Aldrich, #44581). Staining was scored by a single experienced observer, blinded to treatment. Images were acquired on a Hamamatsu NanoZoomer using a 40 X objective, and tumors were analyzed using QuPath analysis software (*14*). Antibodies: Phospho-Src Family (Tyr416) Rabbit CST 2101, 1 / 200; anti-CD3 Rabbit Abcam ab5690, 1/100 and anti-B220 Rat Biolegend 103202, 1/500.

Transcriptomics

RNA was extracted using the RecoverAll Total Nucleic Acid Isolation kit (#AM1975, Ambion) according to the manufacturer's instructions with a DNase digestion step included. 300ng of purified RNA was analyzed using the nanoString PanCancer Immune Profiling Panel following manufacturer's instructions. Hybridization was performed for 18 h at 65 °C prior to processing on the nCounter Prep Station set to high sensitivity. Images were captured (555 Fields of View) and analysed on the nCounter Digital analyser, and data normalized using nSolver 4.0 software.

SUPPLEMENTARY TABLES

Table S1. Diffraction and refinement statistics.

	SRC-eCF506
Space group	$P2_1$
**Cell dimensions**	
a, b, c (Å)	41.8, 62.6, 51.4
α, β, γ (°)	90.0, 98.0, 90.0
Resolution (Å)*	1.50
R_{merge}* (%)	6.7 (76.3)
R_{meas}*(%)	8.0 (91.1)
R_{pim}* (%)	4.3 (49.3)
CC (1/2)*	0.998 (0.626)
Mean (I/σ(I))*	11.7 (1.7)
Completeness (%)*	99.4 (99.4)
Multiplicity *	3.3 (3.3)
**Refinement**	
Resolution (Å)	41.41-1.50
No. reflections	39589
R_{work}/R_{free}	18.0/21.3
No. atoms (all)	2477
Protein	2213

Ligand	41
Water	223
B- factors (all)	19.1
Protein	18.4
Ligand	13.4
Water	26.9
R.m.s. deviation	
Bond lengths (Å)	0.010
Bond angles (°)	1.612

* Highest resolution range shown in parentheses

Table S2. Hydrodynamic radius of SRC (84-536) obtained by SV-AUC and DLS in the absence (SRC-Apo) and presence of dasatinib or eCF506. All values are the mean from 3 measurements. Error = ± S.D.

**Sample**	**SV-AUC** $s_{20,w}$	**DLS** R (nm)
SRC-Apo	3.651±0.004	3.257±0.132
SRC-Dasatinib	3.523±0.021	3.649±0.022
SRC-eCF506	3.656±0.014	3.332±0.042

Table S3. IC_{50} (nM) values calculated for eCF506 and dasatinib in a selection of 16 recombinant kinases. Member of SRC family of kinases are highlighted in red.

	**IC_{50} values (nM)**	
**Kinase**	**eCF506**	**Dasatinib**
**ABL**	479	< 0.5
**BLK**	5.4	< 0.5
**BRK**	17.3	41.5
**FGR**	< 0.5	< 0.5
**FRK/PTK5**	2.8	< 0.5
**FYN**	2.1	< 0.5
**HCK**	2.8	2.0
**KIT**	6,460	39.0
**LCK**	< 0.5	< 0.5
**LYN**	0.8	< 0.5
**mTOR**	>10,000	>10,000
**PDGFRα**	>10,000	9.9
**SRC**	< 0.5	< 0.5
**RET**	>10,000	433
**YES**	< 0.5	< 0.5
**IC_{50} (ABL) / IC_{50} (SRC)**	**950**	**1**
**IC_{50} (KIT) / IC_{50} (SRC)**	**12,920**	**78**

Table S4. Kinome screening of eCF506. Table shows all 340 tested kinases in order of sensitivity to eCF506. Values show the percentage of remaining kinase activity when treated with 1 μM eCF506 at 10 μM of ATP, relative to DMSO. Data represents average of n=2.

Kinase	*% Activity*	*Kinase*	*% Activity*	*Kinase*	*% Activity*	*Kinase*	*% Activity*
SRC	0.1	MAP4K5	81.7	CK2a	92.8	PKG1a	96.6
BRK	0.2	RSK4	83.3	ROCK2	92.8	LRRK2	96.6
HCK	0.8	SGK3	83.4	CDK2/cyclin A1	92.9	CAMK1d	96.7
FGR	1.3	ZAK	83.4	DYRK3	93.0	PKCg	96.7
LYN	1.3	FGFR3	84.7	DAPK3	93.0	FLT3	96.8
LCK	1.4	ALK1	85.9	EPHB3	93.1	TAOK2	96.9
ARAF	1.7	EPHA1	86.7	NLK	93.1	ULK3	97.0
YES	1.8	MAP4K3	87.0	SRPK1	93.3	MAPKAPK2	97.0
LYN B	2.0	PAK3	87.3	CK1e	93.4	RSK2	97.1
FYN	2.3	MET	87.5	SIK2	93.6	CDK1/cyclin A	97.1
FRK	2.4	IKKa	87.6	VRK2	93.6	NEK9	97.1
BMX	2.8	MUSK	89.0	PRKG2	93.7	CDK3/cyclin E	97.2
BTK	3.8	LATS2	89.0	IKKe	93.8	TLK1	97.3
ERBB4	4.5	PKCb2	89.1	ULK1	93.9	MAP4K4	97.3
BLK	4.8	GSK3a	89.2	TESK1	94.3	MLK2	97.3
CSK	5.0	ERK7	89.4	DYRK1B	94.6	MST2	97.4
TXK	9.1	TYK2	89.6	GSK3b	94.6	p38-beta	97.4
RIPK3	12.1	BMPR2	89.6	MAPKAPK5	94.6	CAMK1a	97.4
RAF1	19.6	WNK3	90.3	PKG1b	94.6	JAK2	97.5
ARG	28.7	CDK16/cyclin Y	90.4	FGFR4	94.9	PDPK1	97.5
LIMK2	30.0	PIM3	90.6	MSSK1	94.9	p38-alpha	97.5
MER	30.1	MAP4K2	90.6	TAOK1	95.0	NEK2	97.7
BRAF	35.5	CAMK1b	90.7	JNK3	95.1	MAPKAPK3	97.7
TEC	43.9	RON	90.9	ARK5	95.1	PKCd	97.8
SRMS	46.2	p38-gamma	90.9	HIPK2	95.2	MNK1	97.8
ACK1	54.3	CK1g2	90.9	HIPK3	95.2	MRCKa	97.9
ABL	56.3	ACVR1B	90.9	CLK3	95.2	NEK3	98.0
AXL	64.1	RPS6KA5	91.1	CDK1/cyclin B	95.3	EPHA7	98.1
EPHB2	66.6	MEK1	91.1	PHKg2	95.3	TNIK	98.1
EPHB4	68.5	LCK2	91.2	DYRK1A	95.3	DYRK2	98.2
PAK2	71.2	CAMK2a	91.5	RPS6KB1	95.4	ALK3	98.2
RSK1	72.2	CAMK1g	91.5	MLK1	95.5	ITK	98.2
PKAcb	74.3	DMPK2	91.5	EPHA8	95.6	CK1g3	98.2
MAP3K8	74.6	IRAK1	91.8	CDK9/cyclin K	95.7	ERK5	98.4
Kit	75.1	EPHA5	92.0	STK16	95.7	PHKg1	98.5
NEK11	75.3	PKN1	92.1	PIM1	95.9	PKN3	98.5
RIPK2	76.8	CLK2	92.1	TRKB	95.9	RET	98.6
FGFR1	78.0	PKCtheta	92.1	STK33	96.2	PRKX	98.6
MEK2	78.8	IRAK4	92.4	WEE1	96.2	GRK7	98.6
DDR1	79.6	LTK	92.4	TAOK3	96.2	MARK4	98.6
LIMK1	79.8	MELK	92.5	BRSK2	96.4	MST4	98.7
MEKK3	80.2	CDK6/cyclin D3	92.6	PKCzeta	96.5	SIK1	98.9
NEK7	81.3	CAMK4	92.7	CDK4/cyclin D3	96.6	SGK1	98.9

Kinase	*% Activity*	*Kinase*	*% Activity*	*Kinase*	*% Activity*	*Kinase*	*% Activity*
CHK2	98.9	INSRR	101.4	SIK3	104.0	MNK2	107.3
CDK5/p25	98.9	PIM2	101.4	GRK1	104.0	FER	107.5
TBK1	99.1	GRK5	101.5	TIE2	104.1	MAP3K12	108.2
AURKB	99.1	ROS1	101.5	AKT3	104.2	RPS6KB2	108.2
RPS6KA4	99.2	CDK2/cyclin E	101.6	TGFBR2	104.3	NEK5	108.5
WNK1	99.2	CK1d	101.6	SYK	104.3	PLK1	108.6
STK39	99.2	PAK6	101.7	JNK2	104.3	FES	108.6
PLK2	99.2	EPHA2	101.7	PDGFRb	104.3	PRKD1	108.7
CK1g1	99.2	PKCiota	101.8	VRK1	104.4	IKKb	109.1
MEKK2	99.3	PKAcg	101.8	LOK	104.5	GRK3	109.2
TRKC	99.3	AURKA	101.9	GRK2	104.5	CDK9/cyclin T1	109.2
GRK4	99.5	JAK1	101.9	TTBK1	104.5	ROCK1	109.7
DAPK2	99.5	CAMK2g	101.9	DAPK1	104.6	FGFR2	109.8
ERK2	99.5	CDK7/cyclin H	101.9	PKA	104.6	MLCK	109.8
MARK2	99.5	SLK	102.0	EPHA3	104.7	MST3	110.0
ASK1	99.6	CDK5/p35	102.0	MST1	105.0	RIPK5	110.0
CHK1	99.7	LATS1	102.1	HIPK4	105.0	TGFBR1	110.2
NEK6	99.7	TAK1	102.1	SNARK	105.0	TYRO3	110.5
MAP4K1	99.8	AURKC	102.2	PRKD3	105.0	PASK	110.6
PKCb1	99.8	CDK2/cyclin A	102.3	PLK3	105.1	PKN2	110.8
PKCepsilon	99.9	DDR2	102.4	CDK6/cyclin D1	105.2	JAK3	111.1
PKCa	99.9	GRK6	102.4	DMPK	105.2	CK1a1	111.4
SRPK2	100.0	ALK	102.5	MYO3b	105.2	IR	111.8
EPHA6	100.1	STK22D	102.5	ERK1	105.2	TSSK3	112.0
MEKK1	100.1	ZAP70	102.5	Haspin	105.3	YSK1	112.2
LKB1	100.1	CAMK2d	102.5	WNK2	105.4	PRKD2	113.0
STK38L	100.1	DCAMKL2	102.5	MYLK2	105.5	FAK	113.5
TRKA	100.1	TNK1	102.6	SGK2	105.6	BRSK1	114.7
CLK1	100.2	PKCeta	102.7	MRCKb	105.7	PDGFRa	115.1
MARK1	100.2	RSK3	102.8	ACVR1	105.7	EPHB1	115.5
MLK3	100.2	NEK4	102.8	NEK1	105.9	STK32C	116.0
HIPK1	100.3	DCAMKL1	102.8	TTBK2	106.0	CAMKK2	116.7
TLK2	100.4	OSR1	103.1	JNK1	106.0	PLK4	116.8
PYK2	100.5	SSTK	103.2	P38-delta	106.0	EGFR	117.3
PAK1	100.5	CSF1R	103.3	CDK1/cyclin E	106.1	STK32B	118.6
IGF1R	100.7	CAMK2b	103.5	AKT1	106.3	VEGFR2	119.5
CLK4	100.7	PAK5	103.5	FLT1	106.3	TSSK2	123.4
MEK3	100.8	STK17A	103.6	MAP2K6	106.3	HER2/ERBB2	126.3
MARK3	101.0	CAMKK1	103.6	EPHA4	106.5	FLT4	127.6
AKT2	101.0	CDK4/cyclin D1	103.7	CDC7/DBF4	106.9	MINK	128.9
MYLK3	101.1	ULK2	103.8	MAP2K4	107.0	STK38	175.7
DYRK4	101.3	PBK	103.9	PAK4	107.0	CK2a2	176.1

Table S5. Comparison between the kinome inhibition profile eCF506, dasatinib and bosutinib. All kinases with less than 35% activity remaining after compound addition are shown. SRC family kinases are highlighted in red and ABL in blue. Data for dasatinib and bosutinib obtained from Anastassiadis *et al.* assay (*12*). Note: eCF506 was screened at 1 μM, while dasatinib and bosutinib were tested at 500 nM.

	**eCF506**		**Dasatinib**		**Bosutinib**	
No.	*Kinase*	*% Activity*	*Kinase*	*% Activity*	*Kinase*	*% Activity*
1	SRC	0.1	FRK	-0.9	MAP4K4	1.0
2	BRK	0.2	EPHB2	-0.2	MINK	1.2
3	HCK	0.8	DDR2	0.2	ERBB4	1.5
4	FGR	1.3	EPHA5	0.2	MAP4K5	1.7
5	LYN	1.3	LCK	0.3	ABL	1.8
6	LCK	1.4	LYN	0.4	LCK	1.9
7	ARAF	1.7	CSF1R	0.8	SIK2	1.9
8	YES	1.8	HCK	0.8	LYN	2.1
9	LYN B	2.0	EPHB3	1.0	ARG	2.2
10	FYN	2.3	EPHB4	1.2	MAP4K2	3.2
11	FRK	2.4	TXK	1.4	ACK1	3.5
12	BMX	2.8	LYN B	1.6	EGFR	3.8
13	BTK	3.8	EPHA4	1.6	EPHA6	4.4
14	ERBB4	4.5	FGR	1.7	BTK	5.7
15	BLK	4.8	PDGFRa	1.8	MST4	5.9
16	CSK	5.0	SIK2	1.9	LYN B	6.0
17	TXK	9.1	YES	1.9	SRC	6.0
18	RIPK3	12.1	FYN	2.0	YES	7.3
19	RAF1	19.6	BTK	2.1	MST1	7.4
20	ARG	28.7	PDGFRb	2.5	MEK2	8.4
21	LIMK2	30.0	BMX	2.5	NEK2	8.6
22	MER	30.1	ACK1	2.6	EPHA2	9.6
23	BRAF	35.5	EPHA1	2.7	PAK3	9.9
24	TEC	43.9	ARG	2.7	YSK1	10.1
25	SRMS	46.2	ABL	2.8	EPHA8	10.5
26			BLK	3.0	CK1d	10.9
27			MAP4K5	3.1	MST3	11.0
28			pdg	3.3	ARK5	11.4
29			EPHA3	3.5	CK1e	11.4
30			SRC	3.5	FGR	11.5
31			ERBB4	4.0	PAK1	11.7
32			EPHA8	4.1	MAP3K3	11.8
33			BRK	4.6	EPHB1	11.8
34			EPHB1	4.8	HCK	11.9
35			EPHA2	6.8	CSK	12.3
36			CSK	7.1	EPHB4	12.5
37			ZAK	8.6	LOK	12.6
38			TEC	11.3	MLK3	13.5
39			ALK1	15.9	MST2	13.9
40			LIMK1	16.7	BMX	14.5
41			SRMS	17.0	NEK1	15.2
42			EGFR	21.1	TRKC	15.2
43			NEK11	21.7	MLK1	16.5

44			RIPK2	25.2	CAMK1d	16.6
45			NLK	27.5	EPHB2	17.4
46			ARAF	33.8	RET	18.9
47			MAPK14	36.53	BLK	20.7
48			RAF1	39.66	SYK	21.1
49			RET	45.99	SLK	21.6
50			MINK1	46.54	CHK2	21.7
51			MAP4K4	49.20	MLK2	22.9
52					MAP3K2	23.5
53					MELK	25.8
54					EPHA4	27.0
55					TXK	27.5
56					TAK1	28.3
57					MER	28.9
58					FRK	29.2
59					FYN	29.4
60					LRRK2	30.1
61					FLT3	30.3
62					EPHA3	31.7
63					MYLK2	32.1
64					PYK2	32.6
65					NEK4	32.6
66					ERBB2	32.9
67					PRKD2	35.54
68					FES/FPS	35.71
69					IRR/INSRR	36.90
70					EPHA5	37.14
71					AXL	37.63
72					CHK1	38.90
73					PKCd	40.27
74					MYO3b	40.28
75					WEE1	40.47
76					PRKD3	42.25
77					FMS	43.09
78					PRKD1	43.71
79					FLT4	44.10
80					RSK3	44.20
81					FLT1	44.54
82					TYRO3	47.75
83					EPHB3	49.42
84					PKCa	49.46
85					CK1a1	49.52

Table S6. Summary of clinical scores in acute toxicity study in Swiss mice. Clinical scores describe the number of adverse effects observed at different time points. Vehicle group received the same vehicle concentration as eCF506-treated mice. No clinical signs were recorded for mice treated with 200 mg/kg during this observation period. Clinical signs were observed at 300 mg/kg only for the male group, including slight drop in body temperature and presence of blood in the perianal region. Slight loss of body weight was recorded for 2/3 male mice and one mouse presented hypoactivity. Necropsy examination revealed hyperemia of the digestive track only in male animals. Interestingly, the female group showed no clinical signs after a single dose at 300 or 400 mg/Kg, which may indicate that exposure to eCF506 is slightly different in male and female mice. The single oral administration study in mice concluded: 200 mg/Kg < MTD < 300 mg/kg for male and MTD > 400 mg/Kg for female.

Group	Sex	Female												Male								
	Dose (mg/kg)	Vehicle			200			300			400			Vehicle			200			300		
Time (h)	0	0	0	0	0	0	0	0	0	0	0	0	0	0	0	0	0	0	0	0	0	0
	1	0	0	0	0	0	0	0	0	0	0	0	0	0	0	0	0	0	0	0	0	0
	2	0	0	0	0	0	0	0	0	0	0	0	0	0	0	0	0	0	0	0	2	0
	4	0	0	0	0	0	0	0	0	0	0	0	0	0	0	0	0	0	0	0	3	0
	6	0	0	0	0	0	0	0	0	0	0	0	0	0	0	0	0	0	0	0	3	0
	8	0	0	0	0	0	0	0	0	0	0	0	0	0	0	0	0	0	0	1	2	1
	24	0	0	0	0	0	0	0	0	0	0	0	0	0	0	0	0	0	0	1	2	0

Table S7. Summary of clinical scores in acute toxicity study in Sprague-Dawley rats. Clinical scores describe the number of adverse effects observed at different time points. Vehicle group 1 received the same vehicle concentration as mice treated with 200 mg/kg or 400 mg/kg eCF506, while vehicle group 2 received the same vehicle concentration as mice treated with 700 mg/kg eCF506. As shown in the table, the study provided similar results in both genders, with no clinical signs after single oral dose at 400 mg/Kg. Animals showed adverse effects only when dosed with 700 mg/Kg of eCF506, clinical signs that were equivalent to those observed in male mice treated with 300 mg/Kg of inhibitor.

Group	Sex	Female									Male								
	Dose (mg/kg)	Vehicle 1		Vehicle 2	200		400		700		Vehicle 1		Vehicle 2	200		400		700	
Time (h)	0	0	0	0	0	0	0	0	0	0	0	0	0	0	0	0	0	0	0
	1	0	0	0	0	0	0	0	0	0	0	0	0	0	0	0	0	0	0
	2	0	0	0	0	0	0	0	4	3	0	0	0	0	0	0	0	2	0
	4	0	0	0	0	0	0	0	4	5	0	0	0	0	0	0	0	3	4
	6	0	0	0	0	0	0	0	1	1	0	0	0	0	0	0	0	1	0
	8	0	0	0	0	0	0	0	1	1	0	0	0	0	0	0	0	2	0
	24	0	0	0	0	0	0	0	1	1	0	0	0	0	0	0	0	1	1

SUPPLEMENTARY FIGURES

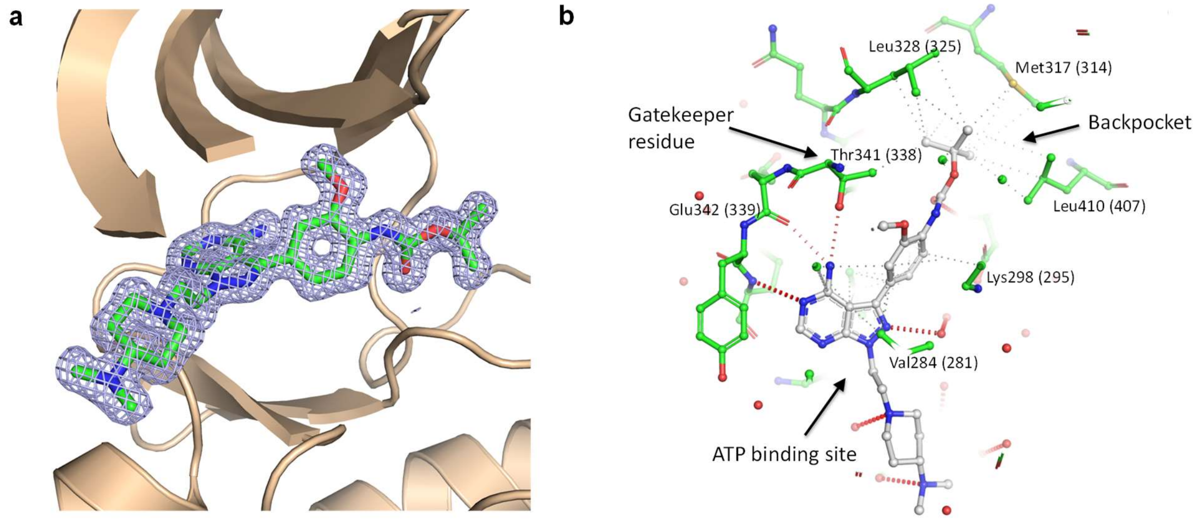

Figure S1. eCF506 binds the ATP binding site of the SRC protein kinase domain in its inactive conformation. (a) 2Fo-Fc Electron density map countered at 1σ (blue mash) is shown around eCF506. As observed in the image, the entire structure of eCF506 is well defined in the electron density maps. **(b)** Main interactions of eCF506 bound to the SRC kinase domain. Red dotted lines = H-bonds. Grey dotted lines = hydrophobic contacts. Amino acid numbering corresponds to human SRC (chicken c-Src numbering given between parentheses). The *tert*-butyl group of eCF506 is deeply buried in a hydrophobic back-pocket only formed in the C-helix-out conformation by M317, L328, I339, F408 and L410. Further, a total of six H-bonds keep a tight grip on eCF506, four of them directly formed with the backbones of E342, M344, D407 and the side chain of T341 and two indirectly via water molecules with the side chain of K298 and the main chain of S348.

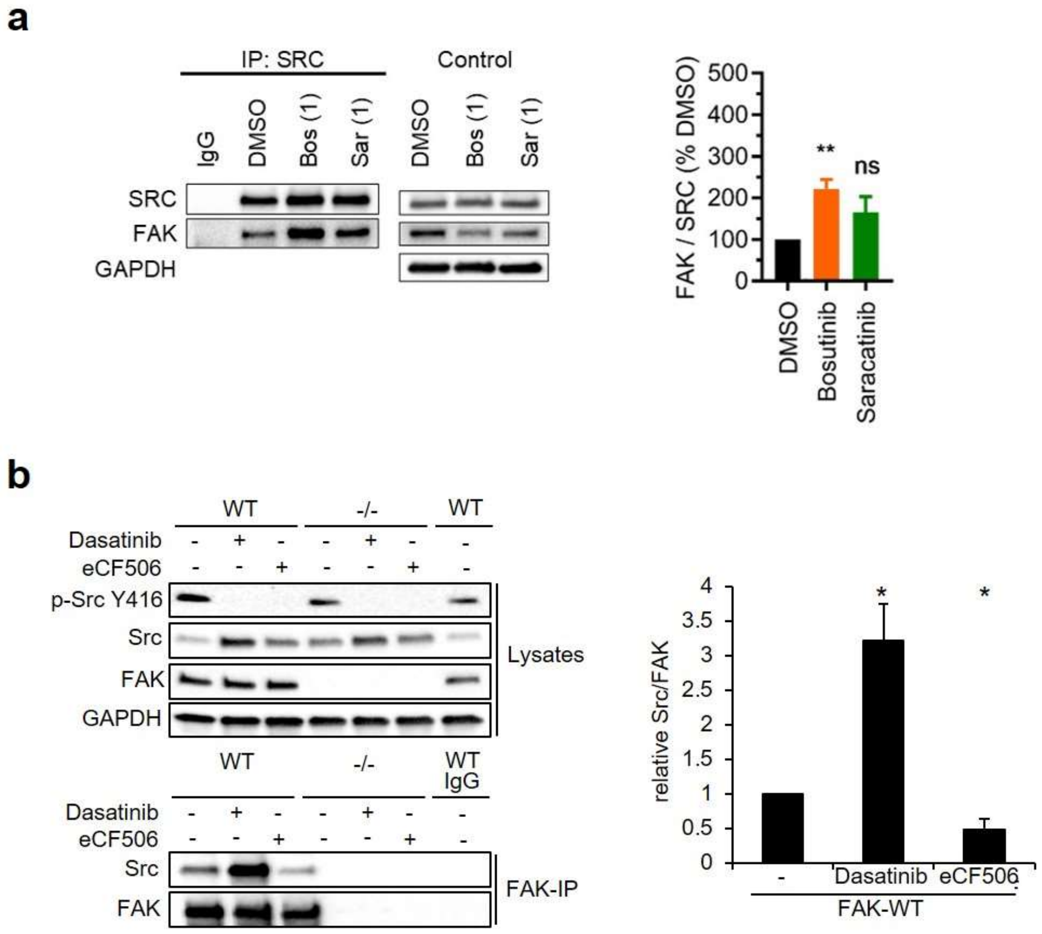

Figure S2. Co-immunoprecipitation studies after drug treatment. (a) MDA-MB-231 cells were treated with bosutinib (1 µM) or saracatinib (1 µM) for 6 h prior to cell lysis and co-immunoprecipitation using SRC antibody overnight. Bound proteins were eluted and analyzed using Western blot with total cell lysates as control. Band intensities were quantified using Bio-Rad ImageLab® software, adjusted for loading by calculating FAK / SRC ratio and normalized to DMSO. Left: Representative Western blots. Right: Summary graph of three biological replicates with standard deviation. ** P <0.01 (t-test). **(b)** Murine SCC cells were treated with dasatinib (0.1 µM) or eCF506 (0.1 µM) following the same protocol as before but co-immunoprecipitating with FAK antibody. Bound proteins were eluted and analyzed using Western blot with cell lysates of SCC FAK-/- cells as a negative control. Left: Representative Western blots. Right: Summary graph showing the quantification of the FAK-IP from three biological replicates with standard deviation. * P <0.05 (t-test).

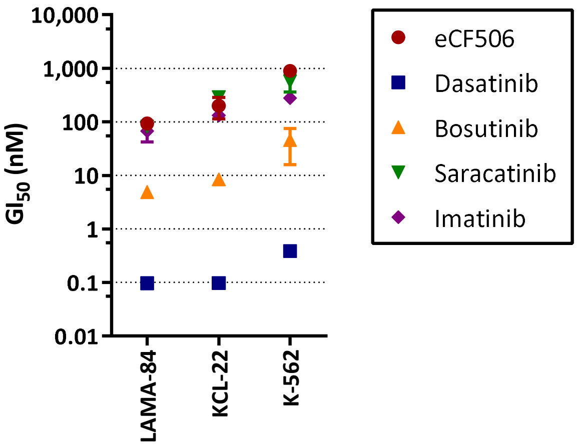

Figure S3. Antiproliferative activities of eCF506, dasatinib, bosutinib, saracatinib and imatinib across three CML cell lines. Viability was tested at day 5 using PrestoBlue. Error bar: ±SD, n=3.

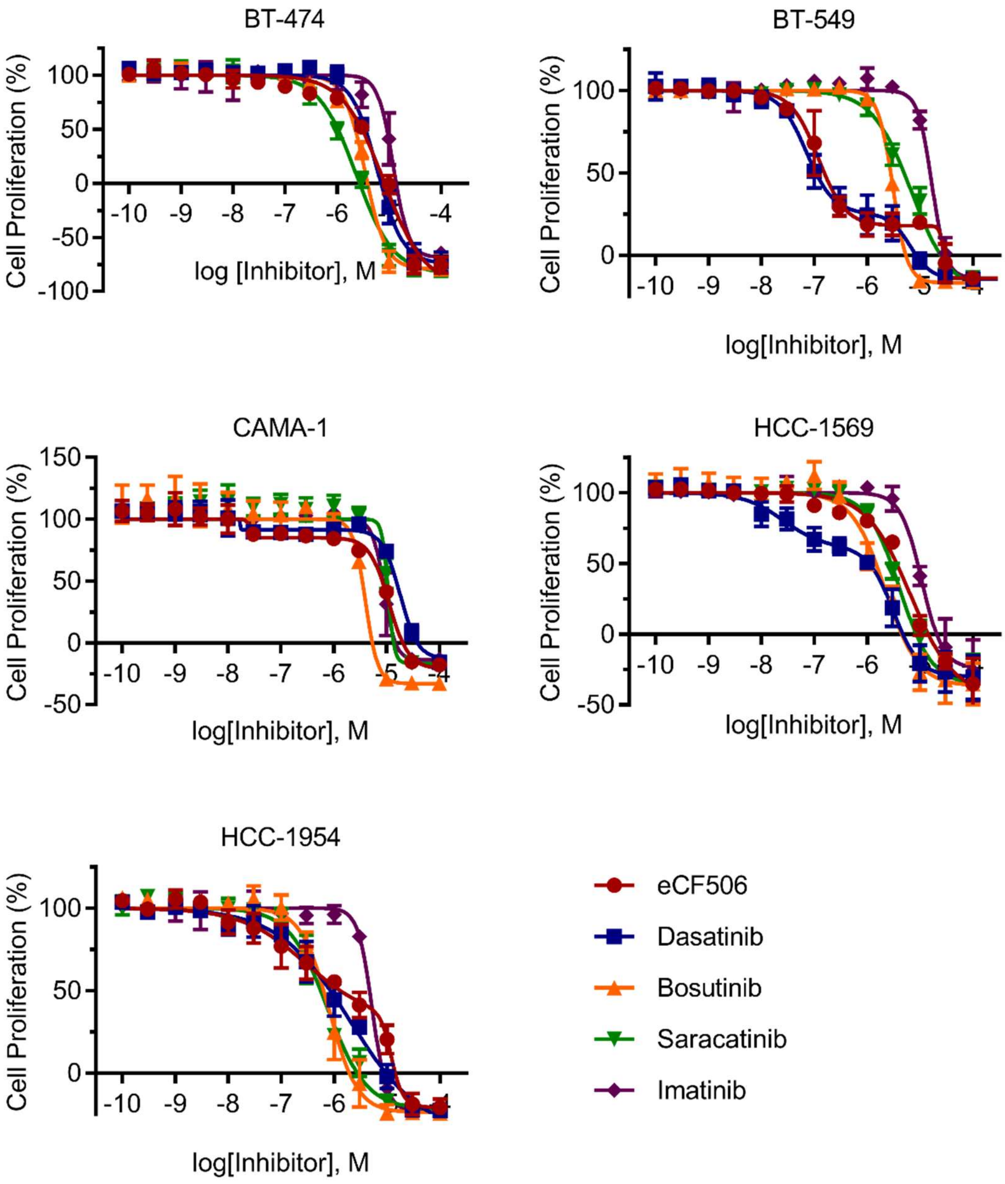

Figure continued on next page

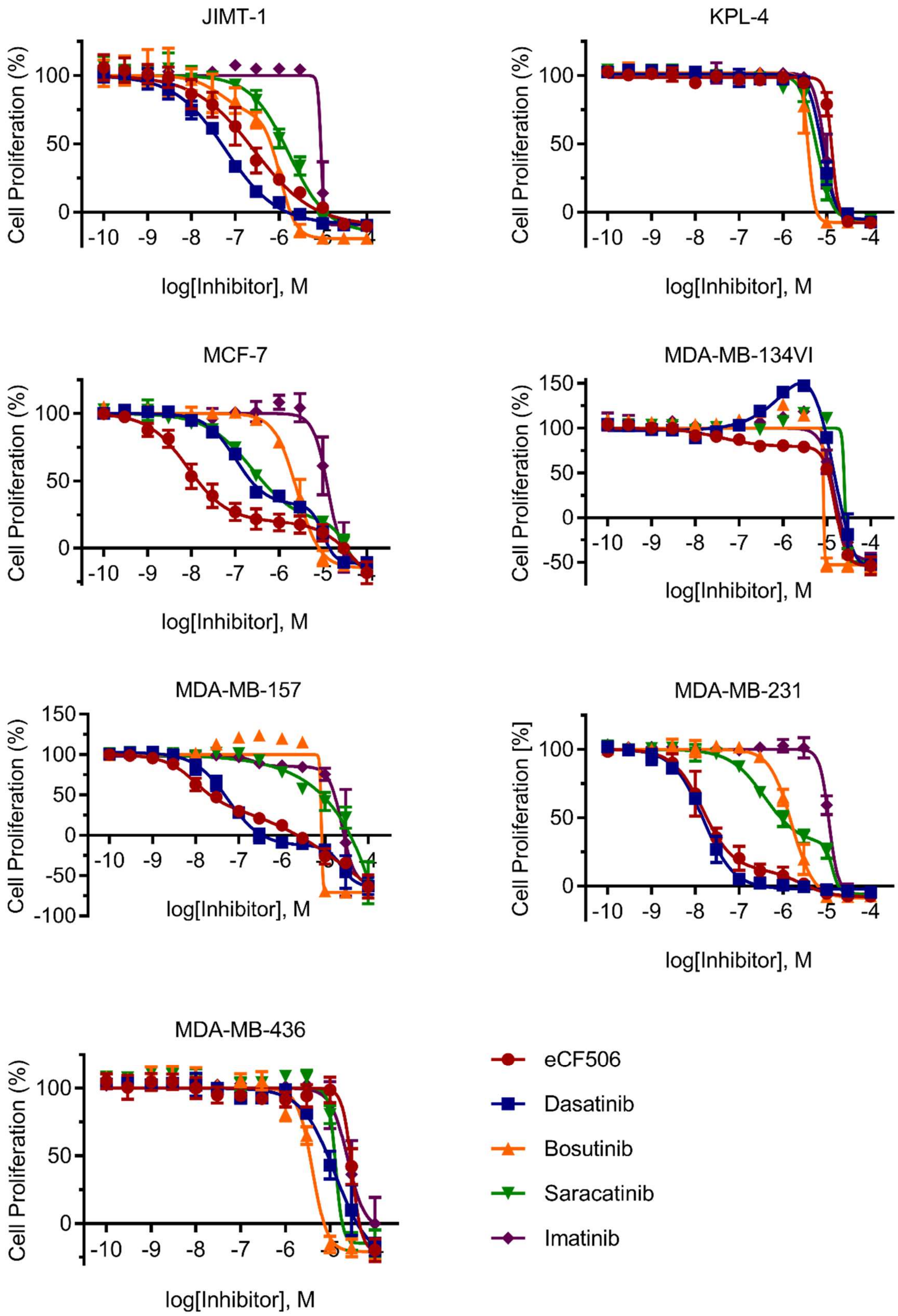

Figure continued on next page

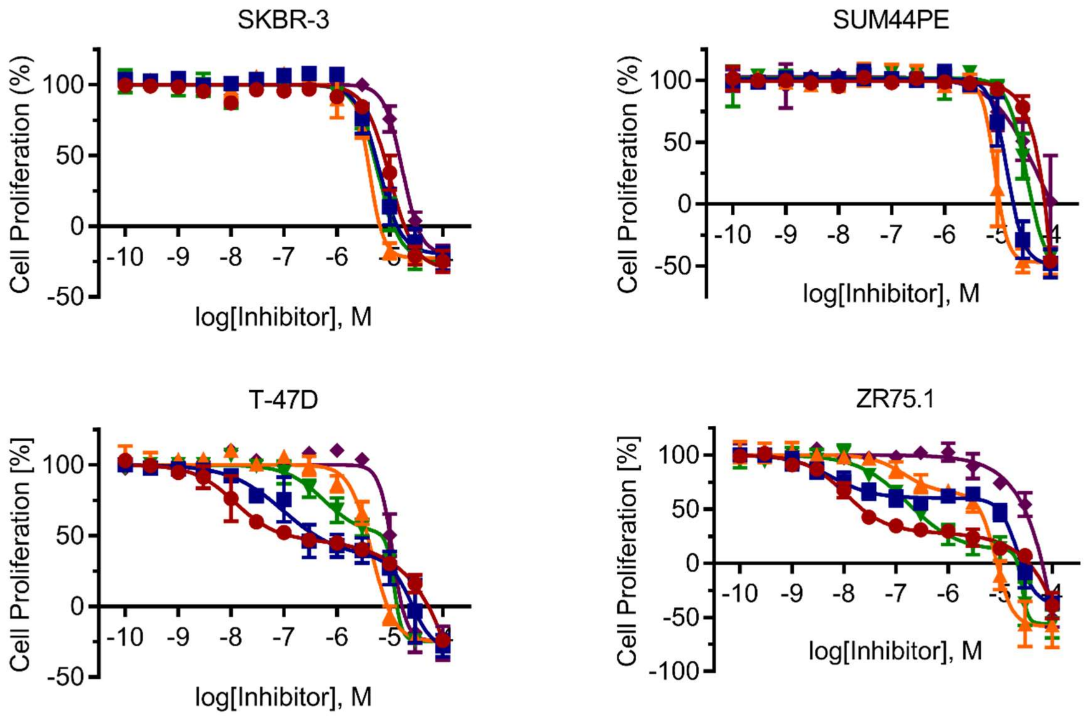

Calculated GI_{50}	Source	Type	Subtype (Subgroup)	eCF506 GI_{50} (in µM)	Dasatinib GI_{50} (in µM)	Bosutinib GI_{50} (in µM)	Saracatinib GI_{50} (in µM)	Imatinib GI_{50} (in µM)
**Breast Cancer**								
**BT-474**	PBr	DC	HER2+ (HER2)	2.99 (0.287)	3.56 (0.430)	2.25 (0.187)	1.05 (0.250)	8.80 (2.46)
**BT-549**	PBr	DC	TNBC (Basal B)	0.155 (0.0564)	0.115 (0.0406)	2.78 (0.159)	5.07 (1.07)	15.7 (2.41)
**CAMA-1**	PE	AC	ER+ (Luminal A)	8.75 (0.464)	15.6 (0.938)	3.49 (0.103)	10.3 (0.831)	8.64 (2.16)
**HCC-1569**	PBr	MC	HER2+ (Basal A-HER2)	3.59 (0.352)	1.17 (0.378)	1.49 (0.422)	2.81 (0.392)	8.84 (1.17)
**HCC-1954**	PBr	DC	HER2+ (Basal A-HER2)	1.79 (0.446)	0.879 (0.341)	0.665 (0.196)	0.568 (0.149)	4.65 (0.204)
**JIMT-1**	PE	DC	HER2+ (HER2)	0.222 (0.141)	0.0484 (0.0119)	0.638 (0.180)	1.33 (0.368)	8.92 (0.929)
**KPL-4**	PE	DC	HER2+ (HER2)	12.8 (1.07)	7.66 (0.643)	3.60 (0.516)	5.79 (0.911)	8.52 (1.64)
**MCF-7**	PE	AC	ER+ (Luminal A)	0.0147 (0.00431)	0.229 (0.0156)	2.26 (0.572)	0.398 (0.0504)	12.7 (4.85)
**MDA-MB-134VI**	PE	LC	ER+ (Luminal A)	10.4 (0.323)	14.1 (2.39)	7.97 (0.148)	24.9 (0.673)	12.1 (2.03)

**MDA-MB-157**	PE	M	TNBC (Basal B)	0.0200 (0.00281)	0.0486 (0.0143)	7.61 (0.189)	6.83 (1.38)	21.3 (14.1)
**MDA-MB-231**	PE	AC	TNBC (Basal B)	0.0160 (0.00498)	0.0169 (0.00350)	1.45 (0.206)	0.983 (0.307)	10.7 (0.284)
**MDA-MB-436**	PE	AC	TNBC (Basal B)	27.0 (3.13)	9.33 (3.67)	3.42 (0.428)	12.6 (1.35)	27.5 (11.2)
**SKBR-3**	PE	AC	HER2+ (HER2)	7.80 (1.21)	5.49 (0.366)	3.65 (0.382)	4.99 (0.402)	15.2 (2.61)
**SUM44PE**	PE	LC	HER2+ (Luminal B)	50.8 (7.66)	12.7 (4.15)	7.87 (2.28)	26.9 (5.81)	39.8 (20.0)
**T-47D**	PE	DC	ER+ (Luminal A)	0.149 (0.0983)	0.497 (0.363)	3.70 (0.500)	3.46 (0.729)	10.4 (1.40)
**ZR75.1**	A	DC	ER+ (Luminal A)	0.0218 (0.00223)	11.2 (5.09)	4.04 (0.693)	0.248 (0.0687)	28.7 (3.90)

Figure S4. Dose-response curves and calculated GI_{50} values. Dose-response curves of 16 human breast cancer cell lines treated with different SRC/ABL kinase inhibitors for 5 days. Effects on cell proliferation were measured using PrestoBlue^{TM} reagent and curves were fitted using GraphPad Prism 7. Fluorescence intensity readings on the day of treatment was subtracted from final readings and results were normalized to DMSO treated control. All dose-response curves represent the average of three biological repeats with standard deviation in brackets.

Breast cancer sources: PBr = primary breast, PE = pleural effusion, A = ascites. Breast cancer type: AC = adenocarcinoma, DC = ductal carcinoma, MC = metaplastic carcinoma, M = medullary carcinoma, LC = lobular carcinoma.

Breast cancer subtypes: HER2+ = HER2 amplified, ER+ = oestrogen receptor expressing, TNBC = triple negative breast cancer lacking ER, PrR and HER2.

Breast cancer subgroups: Luminal A (ER+, PR±, HER2-, Ki67 low), Luminal B (ER+, PR±, HER2+, Ki67 high), Basal A (luminal-like), Basal B (basal-like, claudin-low), HER2 (ER-, PR-, HER2 amplified).

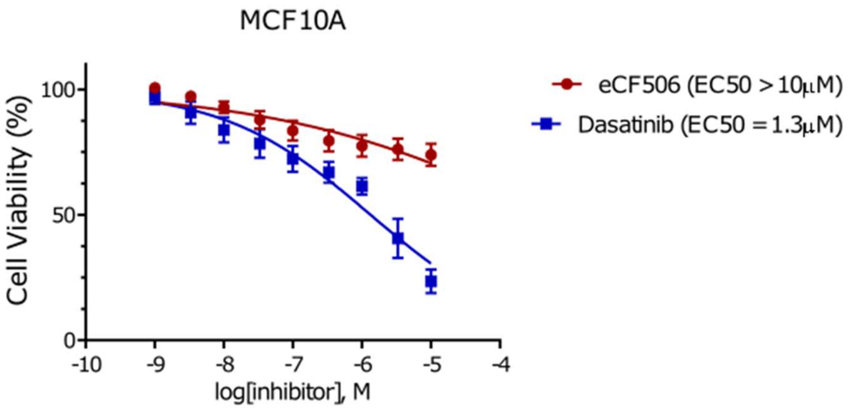

Figure S5. Effect of SRC inhibition on non-malignant breast epithelial cells. Dose-response curves of human non-malignant breast epithelial cell MCF10A after treatment with eCF506 or dasatinib for 5 d. Effects on cell proliferation were measured using PrestoBlue™ reagent and curves were fitted using GraphPad Prism 7. Fluorescence intensity readings on the day of treatment was subtracted from final readings and results were normalized to DMSO treated control. Error bar: ±SD, n=3.

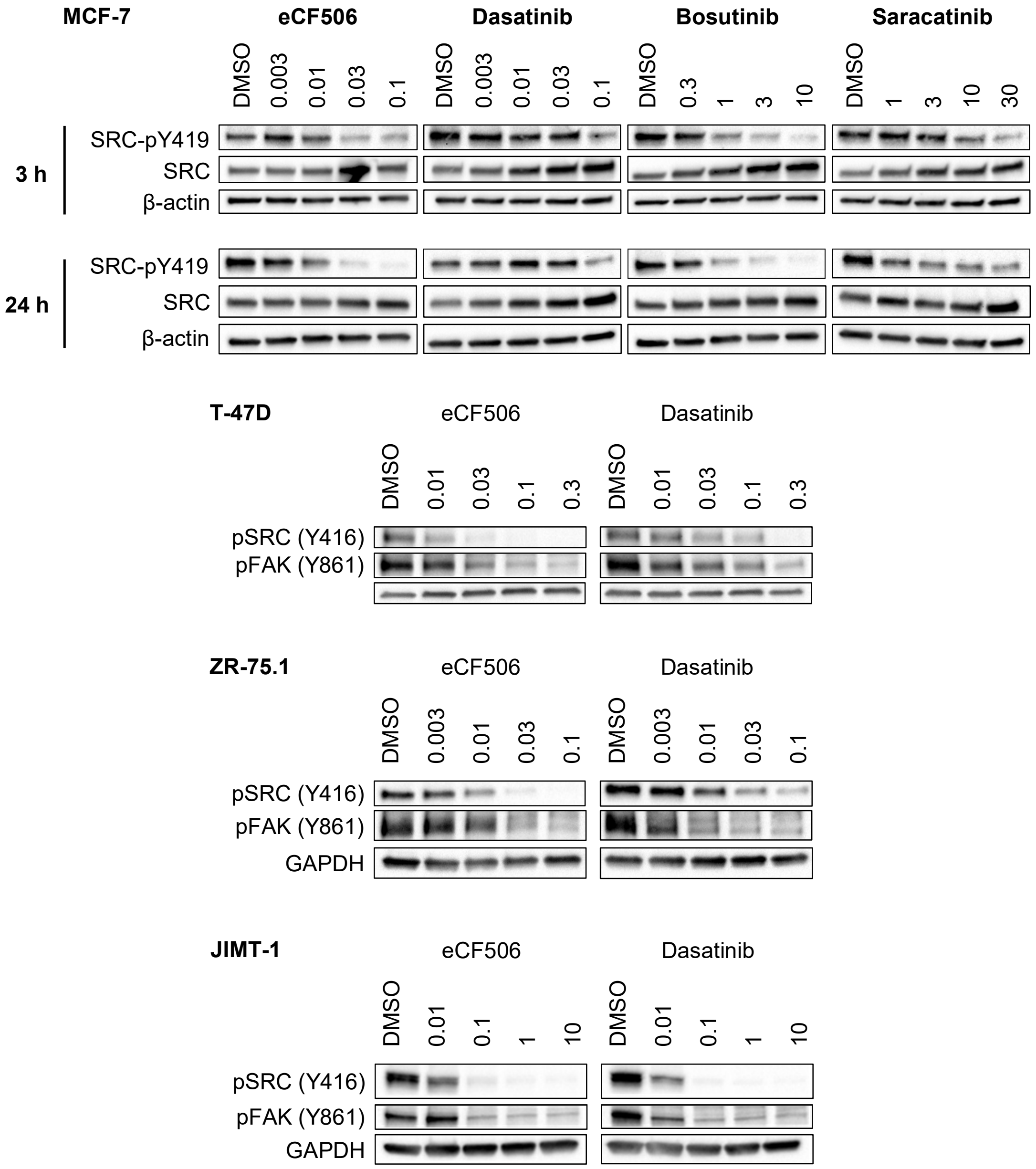

Figure S6. Western blot analysis of eCF506 and clinical SRC inhibitors in ER+ MCF-7, T-47D and ZR-75.1 cells and HER2+ JIMT-1 cells. Cell lines were treated with the inhibitors for 24 h prior to cell lysis and analysis by Western blot. Concentrations are in μM.

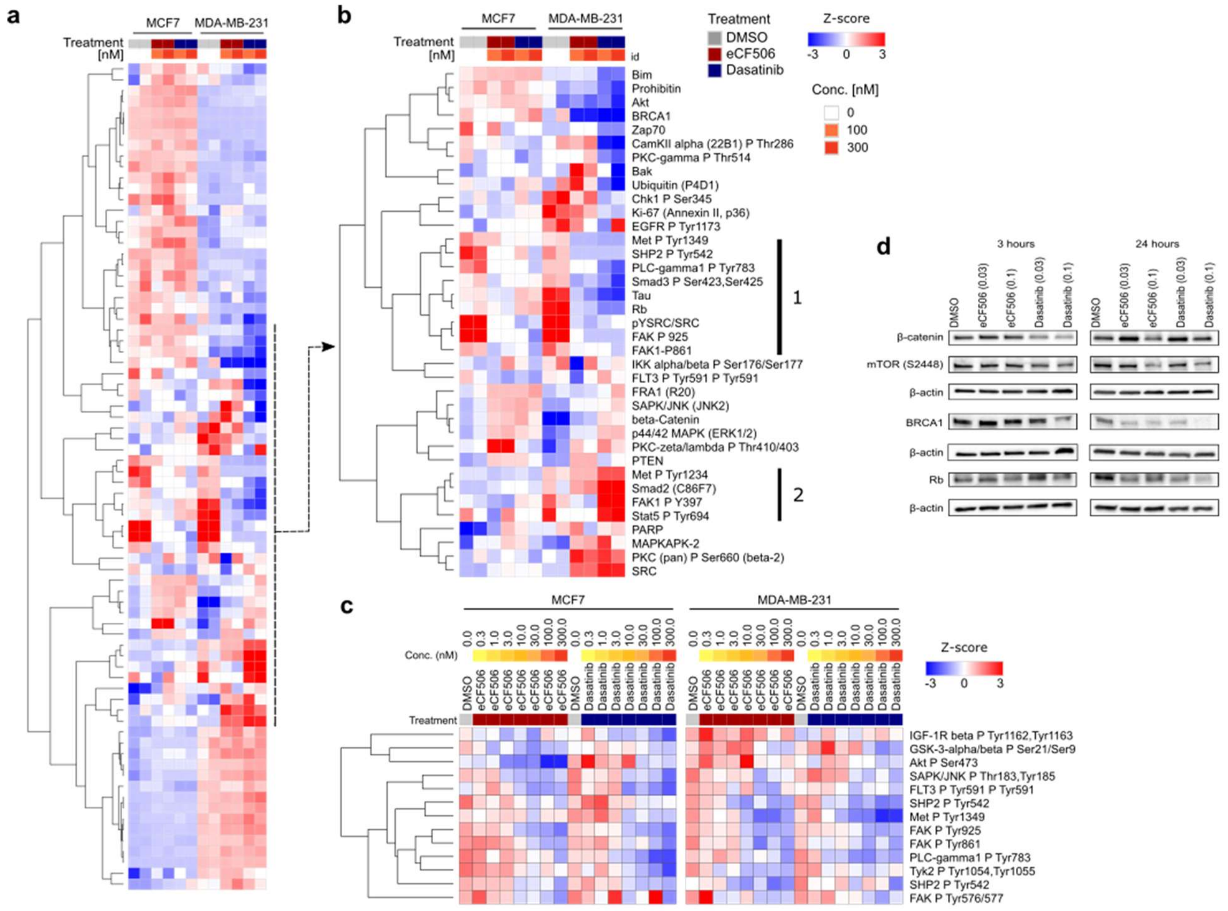

Figure S7. (a,b) Hierarchical clustering analysis of the RPPA study in MCF-7 and MDA-MB-231 cells after 24 h treatment with eCF506 or dasatinib. Most relevant changes and antibody labels identified are compiled in **b**. **(c)** Analysis of 13 phosphorylated proteins after 3 h treatment with eCF506 or dasatinib at a range of concentrations. **(d)** Western blot analysis of selected proteins assessed in the RPPA. MDA-MB-231 cells were treated with eCF506 or dasatinib for the time specified prior to lysis and analysis by Western blot. Images represent one biological replicate. Concentrations given in µM.

Highlights of the proteomics analysis. The MCF-7 and MDA-MB-231 cell lines exhibited characteristic differences in protein expression, for example, no caspase 3 present in MCF-7 cells and no E-cadherin in MDA-MB-231 cells. An overall increase in pro-apoptotic protein BAK and PTEN was observed in MDA-MB-231 cells treated with eCF506 but not with dasatinib, whereas dasatinib increased β-catenin in this cell line and eCF506 did not. Decrease of BRCA1 and Rb proteins was observed in MDA-MB-231 cells after 24 h of treatment with both compounds. Reduced expression of BRCA1 and Rb protein was also observed in MCF-7 cells after treatment with eCF506 (Figure S7b, group 1), but dasatinib increased the levels of both proteins in this cell line. These contrasting effects in proteins that regulate the cell cycle may be related to the superior antiproliferative potency of eCF506 in MCF-7 cells. Increased levels of PKCζ/λ-pT410/403 was also detected in cells treated with eCF506 but not with dasatinib.

Fig. S7c shows dose response changes on 13 phosphorylated proteins that have been proposed to belong to the interaction network of the SRC-FAK axis (*15-21*). Similar change trends were observed for both dasatinib and eCF506, showing the wider inhibition of signaling networks due to SRC inhibition.

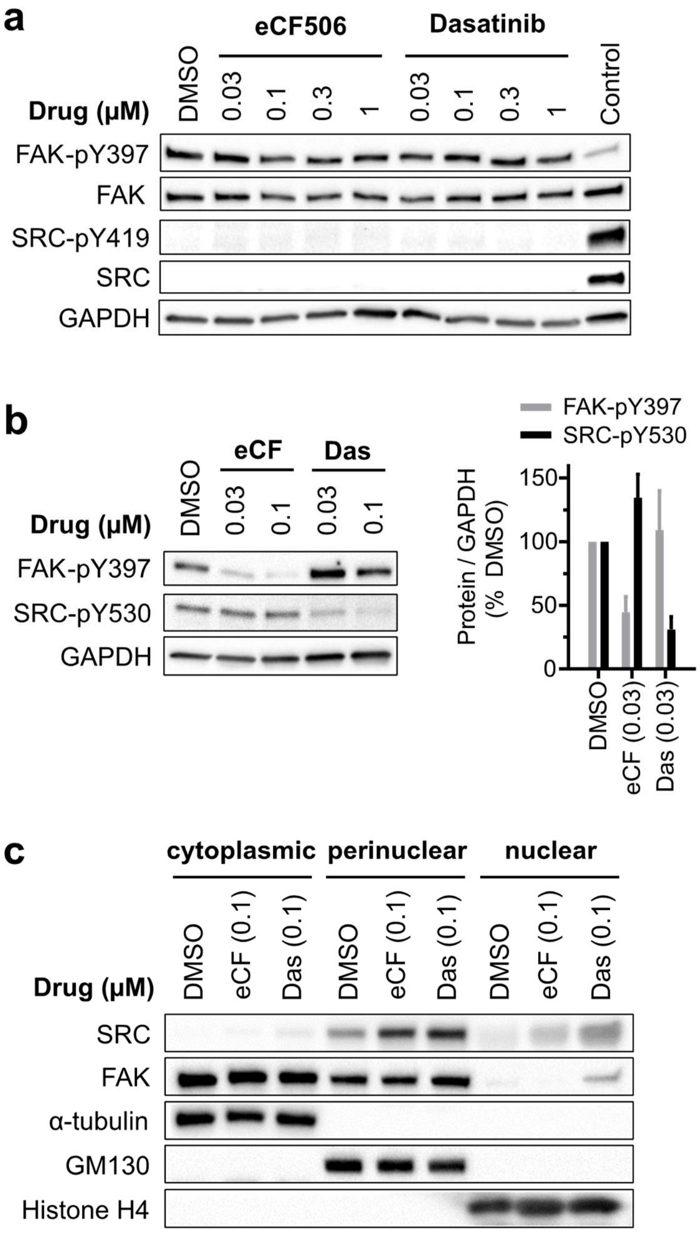

Figure S8. (a) Analysis of FAK autophosphorylation after treating SYF ($SRC^{-/-}$, $YES^{-/-}$, $FYN^{-/-}$) cells for 24 h with eCF506 or dasatinib. DMSO-treated MDA-MB-231 cells and analysis of total SRC and SRC-pY419 were used as controls. Representative Western blots from n = 3. **(b)** Representative Western blots of FAK-pY397 and SRC-pY530 after treating MDA-MB-231 cells with eCF506 or dasatinib for 3 h. Quantitative analysis of FAK-pY397 and SRC-pY530 levels normalized to DMSO after treatment with 0.03 µM of each inhibitor from n = 3 is shown on the right. Error bars: ± SD. **(c)** Subcellular localization study of SRC and FAK in MDA-MB-231 cells after treatment with eCF506 or dasatinib for 6 h. α–tubulin, GM130 and histone H4 were used as subcellular fractionation controls. Representative Western blots from n = 3.

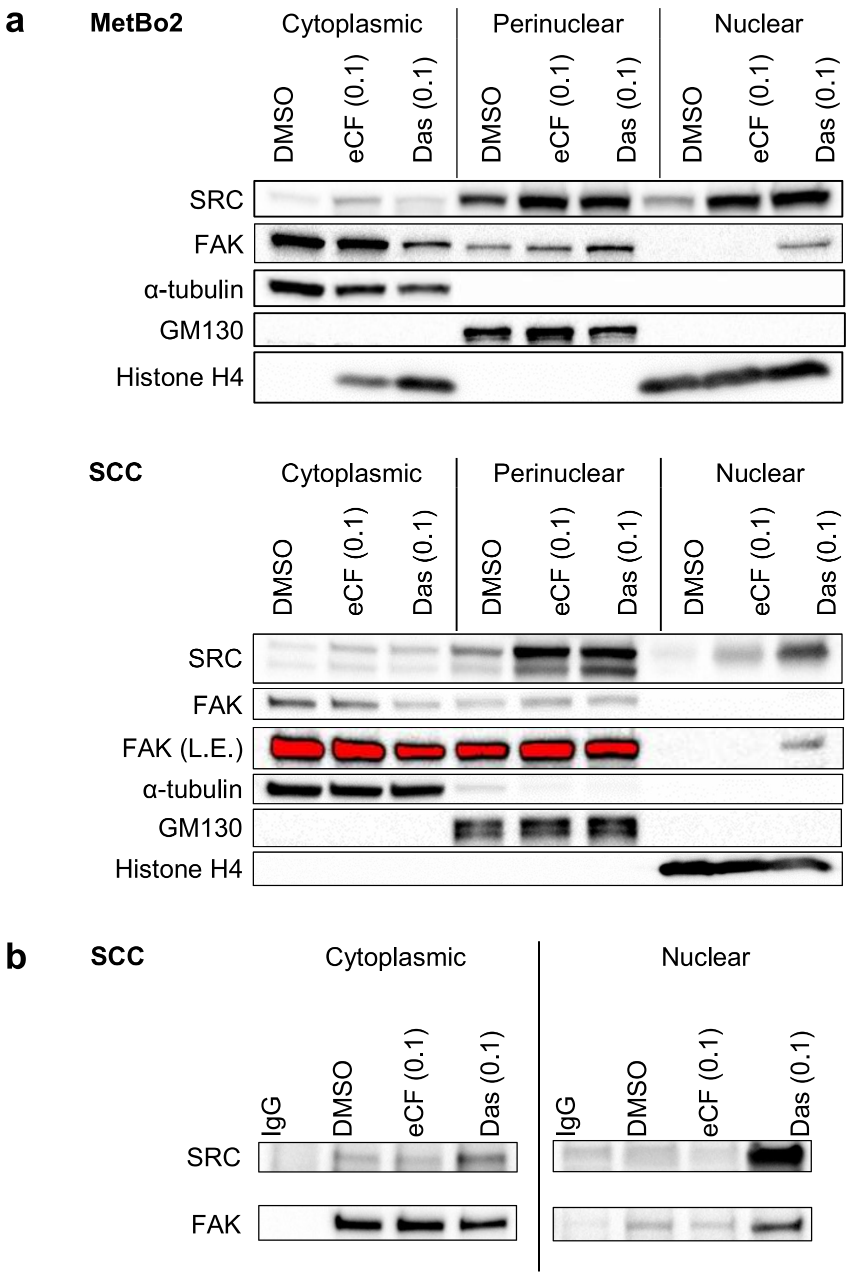

Figure S9. (a) Subcellular fractionation of MetBo2 and SCC cells after treatment with eCF506 or dasatinib (both 0.1 μM) for 6 h prior to subcellular fractionation and analysis by Western blot. Representative Western blot of three biological repeats. (L.E.) = longer exposure. **(b)** Co-immunoprecipitation of FAK in cytoplasmic and nuclear fraction. SCC cells were treated with eCF506 or dasatinib (both 0.1 μM) for 6 h prior to subcellular fractionation followed by co-immunoprecipitation using FAK antibody overnight. Representative Western blot of three biological repeats. All concentrations given in μM.

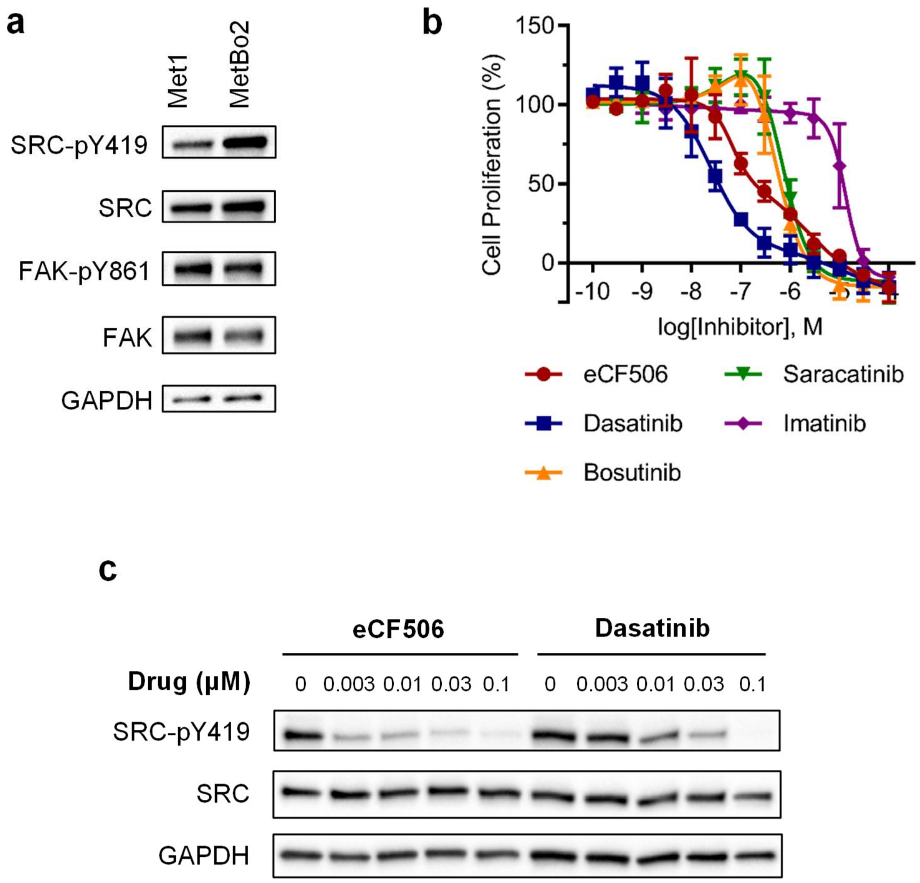

Figure S10. (a) Analysis of total and phosphorylated Src and FAK signature in murine Met1 and MetBo2 cells. Representative Western blot of three biological repeats. **(b)** Cell proliferation of MetBo2 cells treated for 5 d with inhibitors (eCF506, dasatinib, bosutinib, saracatinib and imatinib). Graph shows average of three biological repeats with standard deviation. **(c)** Analysis of total and phosphorylated Src after treating MetBo2 cells with eCF506 and dasatinib. Cells were treated with the inhibitors for 24 h prior to cell lysis and analysis by Western blot.

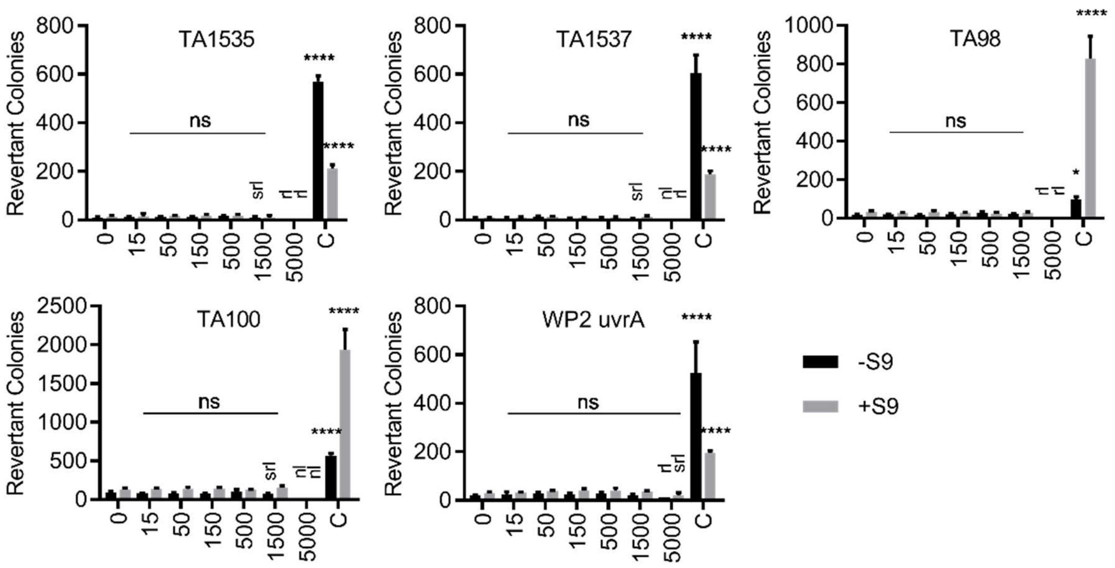

Figure S11. Ames test of eCF506. The mutagenicity of eCF506 was tested in five different auxotrophic bacterial strains. Graphs show average of three biological repeats with standard deviation. Bacteriotoxic effects: srl = slightly reduced lawn, rl = reduced lawn, nl = no lawn. Statistical analysis was done by two-way ANOVA with Dunnett's correction for multiple comparison showed no significant ($P > 0.05$) changes for eCF506, while the controls all significantly increased the number of revertant colonies compared to the respective untreated control; ns, $P > 0.05$; *, $P < 0.05$; ****, $P < 0.0001$. The test produced no greater increases in revertants than those expected from normal variation in the negative control number of revertants, for each strain. Consequently, the test was considered negative, confirming eCF506 is not mutagenic according to this assay.

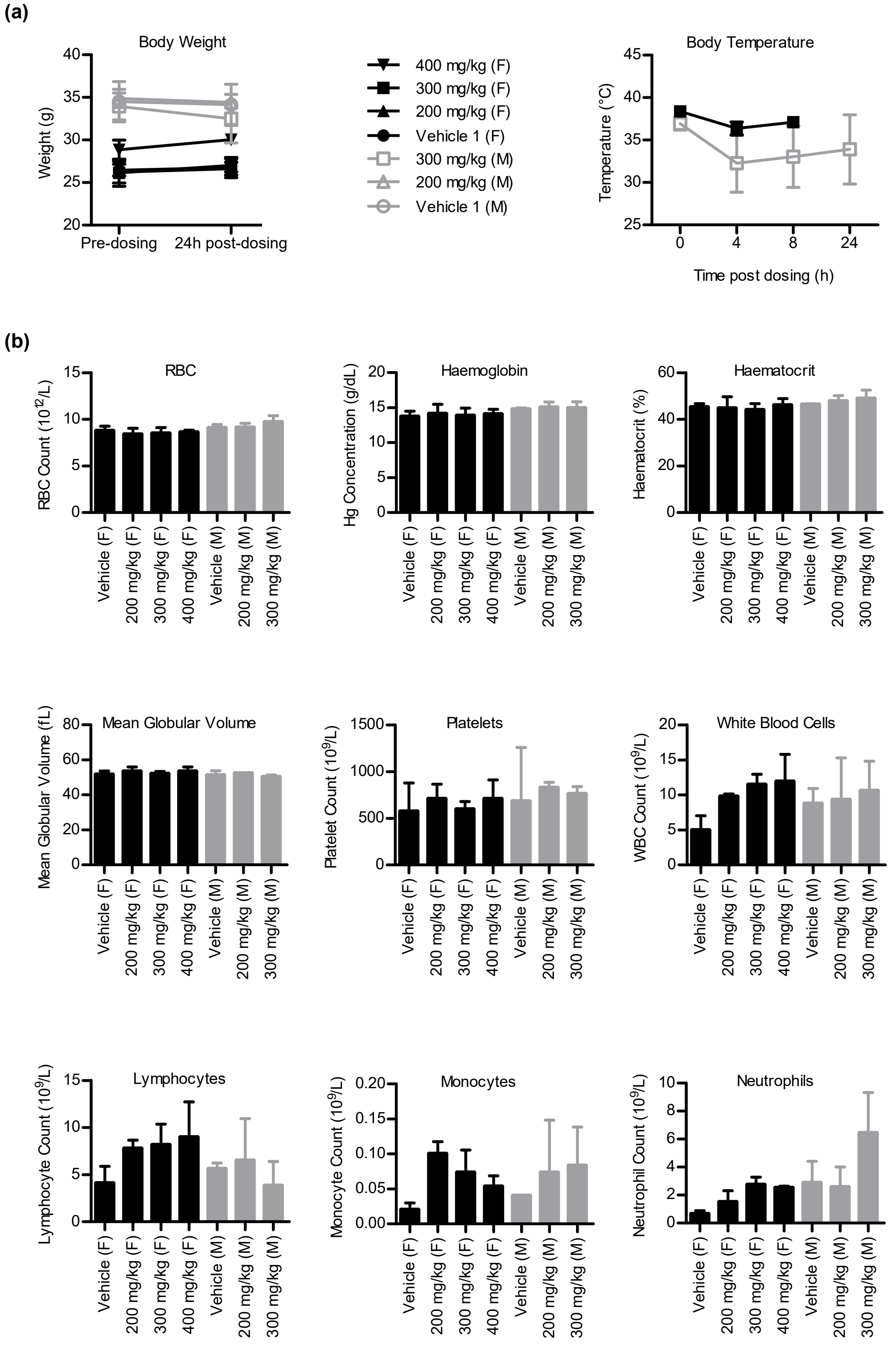

Figure S12. Acute toxicity study. **(a)** Body weight and temperature and **(b)** Haematology results of individual Swiss mice treated with eCF506 in an acute toxicity study. 3 mice/group. Results showed increased white blood cell count and no change in red blood cell count.

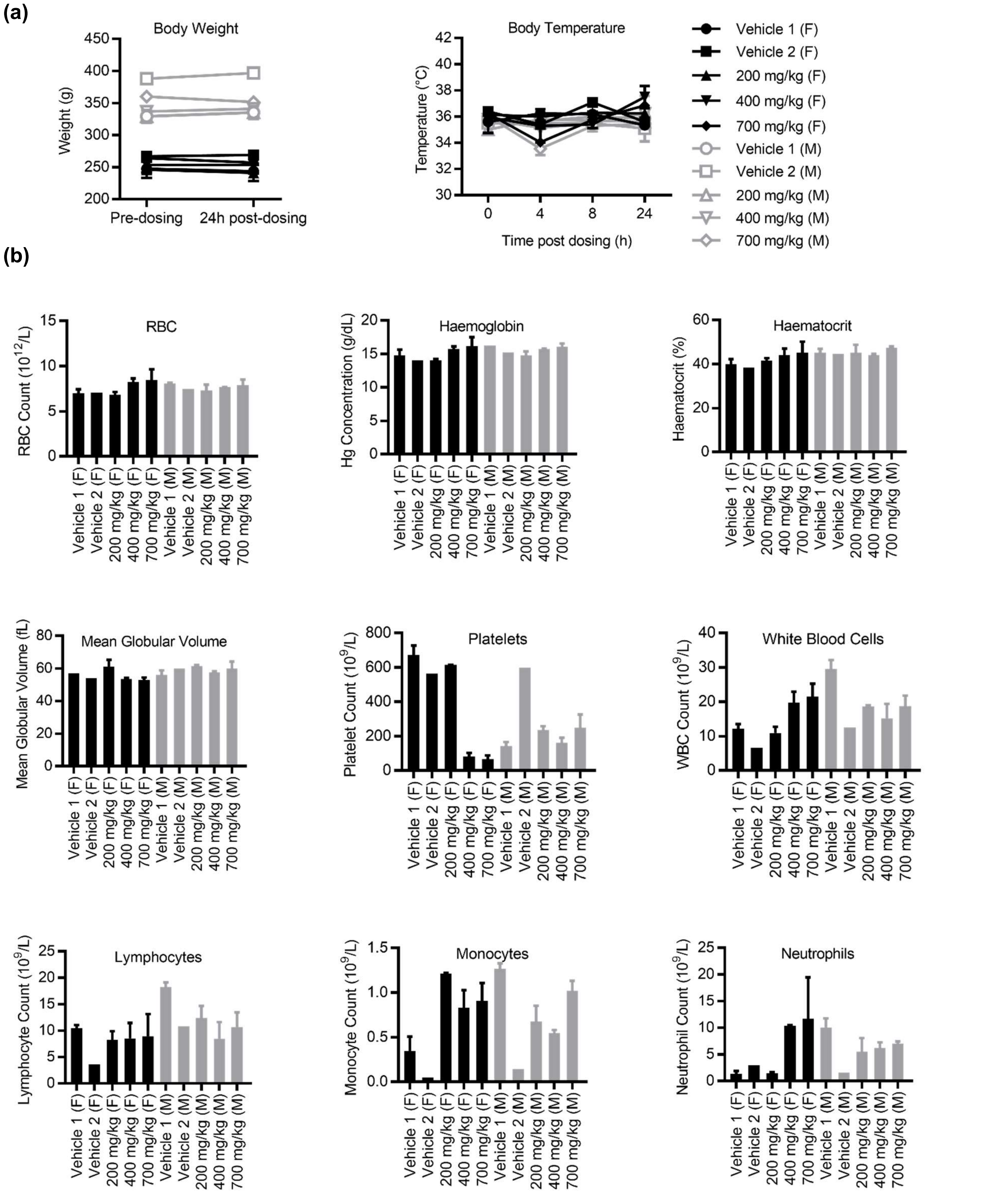

Figure S13. Acute toxicity study. **(a)** Body weight and temperature and **(b)** Haematology results of individual Sprague-Dawley rats treated with eCF506 in an acute toxicity study. No statistical comparison was performed due to small sample size (1-2 rats/group).

a

Time h	Animal ID	Blood nM
2	**10**	3618
	**11**	4238
	**12**	4405
	**MEAN**	**4087**
	**SD**	**415**
8	**13**	969
	**14**	753
	**15**	756
	**MEAN**	**826**
	**SD**	**124**
24	**16**	92.5
	**17**	279
	**18**	244
	**MEAN**	**205**
	**SD**	**99.0**

PK parameters following single oral administration at 40 mg/kg of eCF506 in female CD1 mice						
**Matrix**	**Cmax (ng/mL - ng/g)**	**tmax (h)**	**AUC (0-last) (ng.h/mL)**	**AUC (0-inf) (ng.h/mL)**	**tlast (h)**	**t1/2z (h)**
Blood *(rating)*	2087	2	11979	12810	24	5.5 *(long)*

b

Time h	Animal ID	Blood nM
2	**13**	4732
	**14**	7272
	**15**	8675
	**MEAN**	**6893**
	**SD**	**1999**
8	**16**	1497
	**17**	1665
	**18**	1167
	**MEAN**	**1443**
	**SD**	**253**
24	**19**	351
	**20**	516
	**21**	415
	**MEAN**	**427**
	**SD**	**83.4**

PK parameters following single oral administration at 80 mg/kg of eCF506 in female CD1 mice								
**Matrix**	**Cmax (ng/mL - ng/g)**	**tmax (h)**	**AUC (0-last) (ng.h/mL)**	**AUC (0-inf) (ng.h/mL)**	**Ratio AUClast80 / AUClast40**	**Ratio AUCinf80 / AUCinf40**	**tlast (h)**	**t1/2z (h)**
Blood *(rating)*	3520	2	21016	22897	1.8	1.8	24	6.0 *(long)*

Figure S14. Blood levels of eCF506 in female mice (n = 3 / group) at 2, 8 and 24 h following single oral administration of eCF506 at **(a)** 40 mg/Kg or **(b)** 80 mg/Kg. Calculated PK parameters are shown on the right.

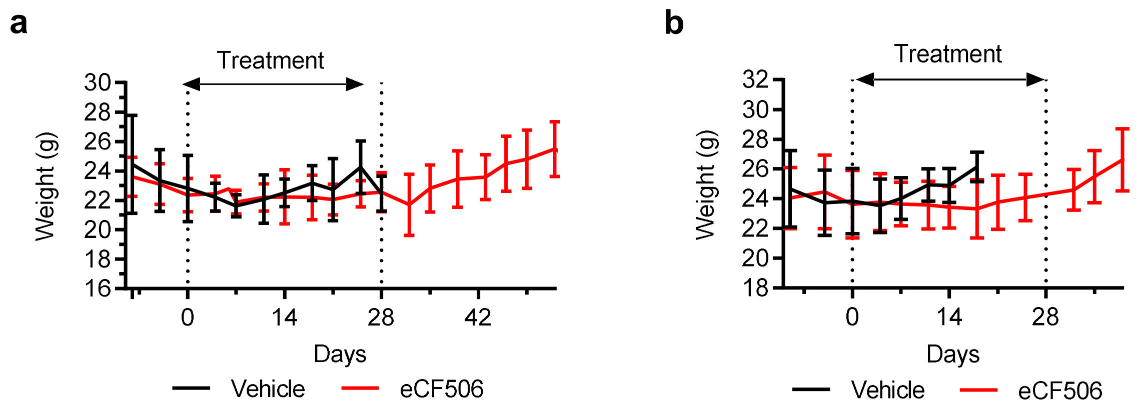

Figure S15. Analysis of animals weight over the course of the study. **(a)** FVB mice. **(b)** CD1 nude mice.

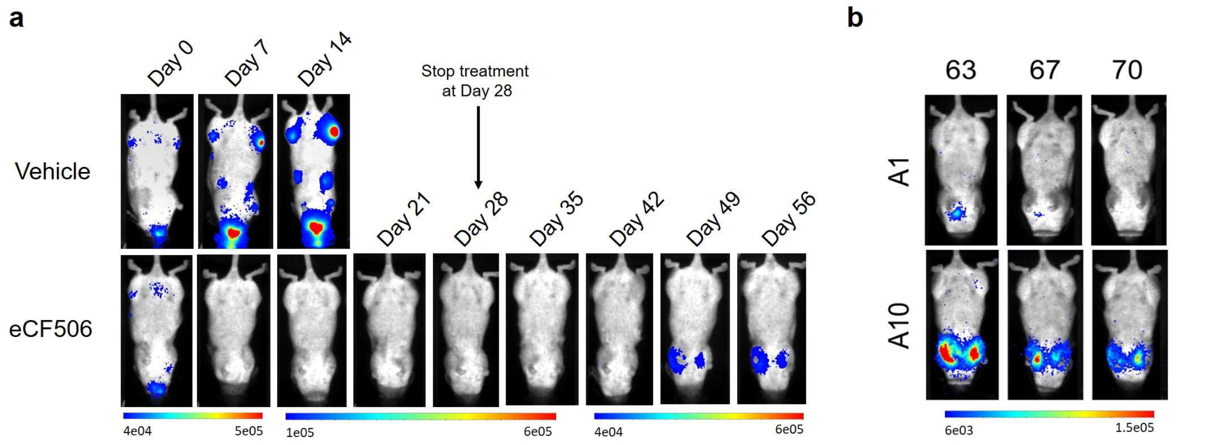

Figure S16. (a) Weekly bioluminescence tomography imaging of one mouse treated with vehicle vs. one mouse (A10) treated with eCF506. Note the formation of lung metastases in the eCF506-treated mouse at day 49. **(b)** Bioluminescence tomography images of two mice (A1 and A10) with relapsed tumors in the thoracic box at day 63. Treatment with eCF506 (40 mg/Kg, QD) was re-started on day 63 for 7 days, showing reduction of metastatic burden in both mice.

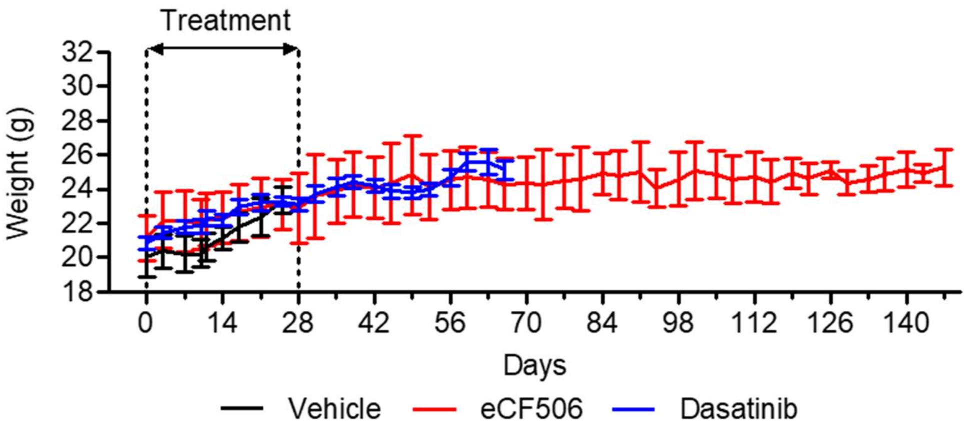

Figure S17. Analysis of animals weight over the course of the head-to-head efficacy study in wildtype FVB mice.

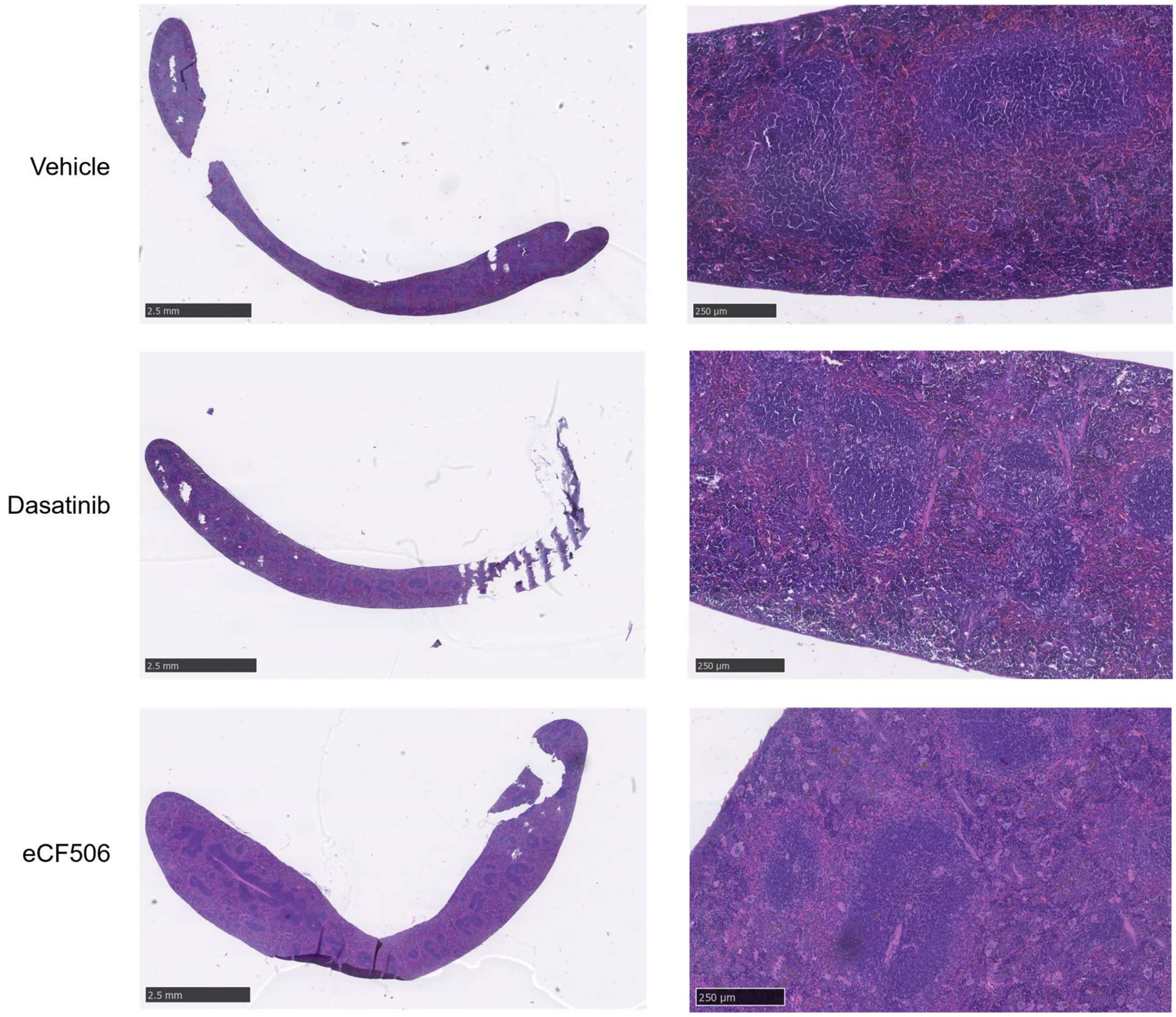

Figure S18. Representative H&E-stained histology images of spleens from tumour-bearing mice treated with vehicle (top), dasatinib (centre) and eCF506 (bottom). Whole spleens are shown at the left (size bar 2.5 mm) for comparison of spleen size and magnified tissue sections on the right (magnification x10, size bar 250 μm), showing normal splenic histology.

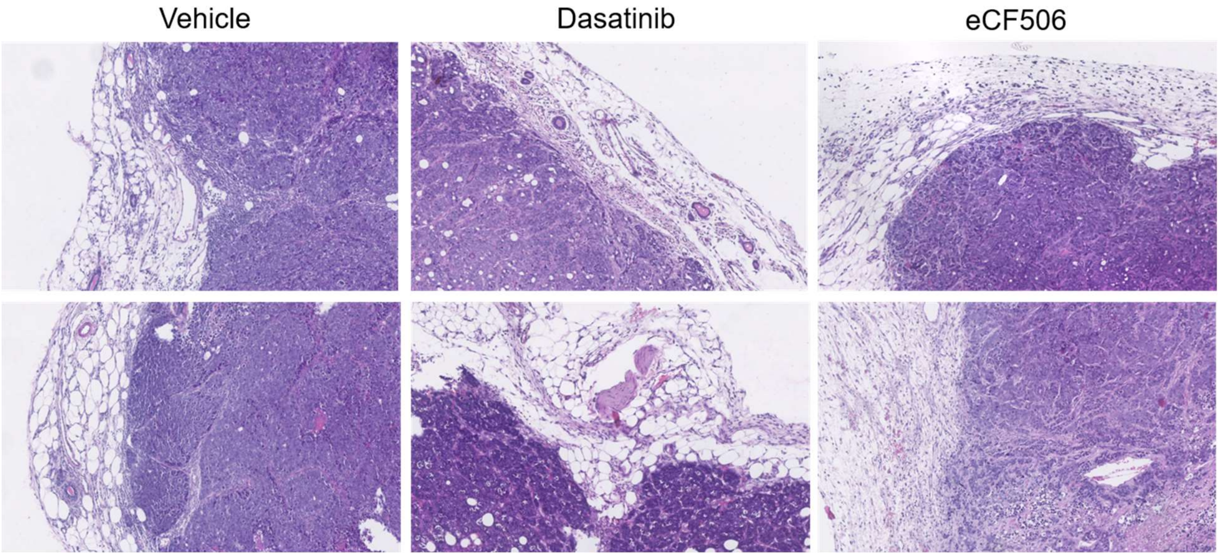

Figure S19. Representative H&E-stained histology images (magnification x10) of MetBo2 orthotopic breast tumors treated with vehicle (left), dasatinib (centre) and eCF506 (right). Images illustrate the peritumoral area with surrounding adipose-rich connective tissue showing differences between the groups in peritumoral chronic inflammation and lymphocyte infiltration (moderate to marked for the eCF506-treated tumors, but only mild for the vehicle control and dasatinib-treated tumors).

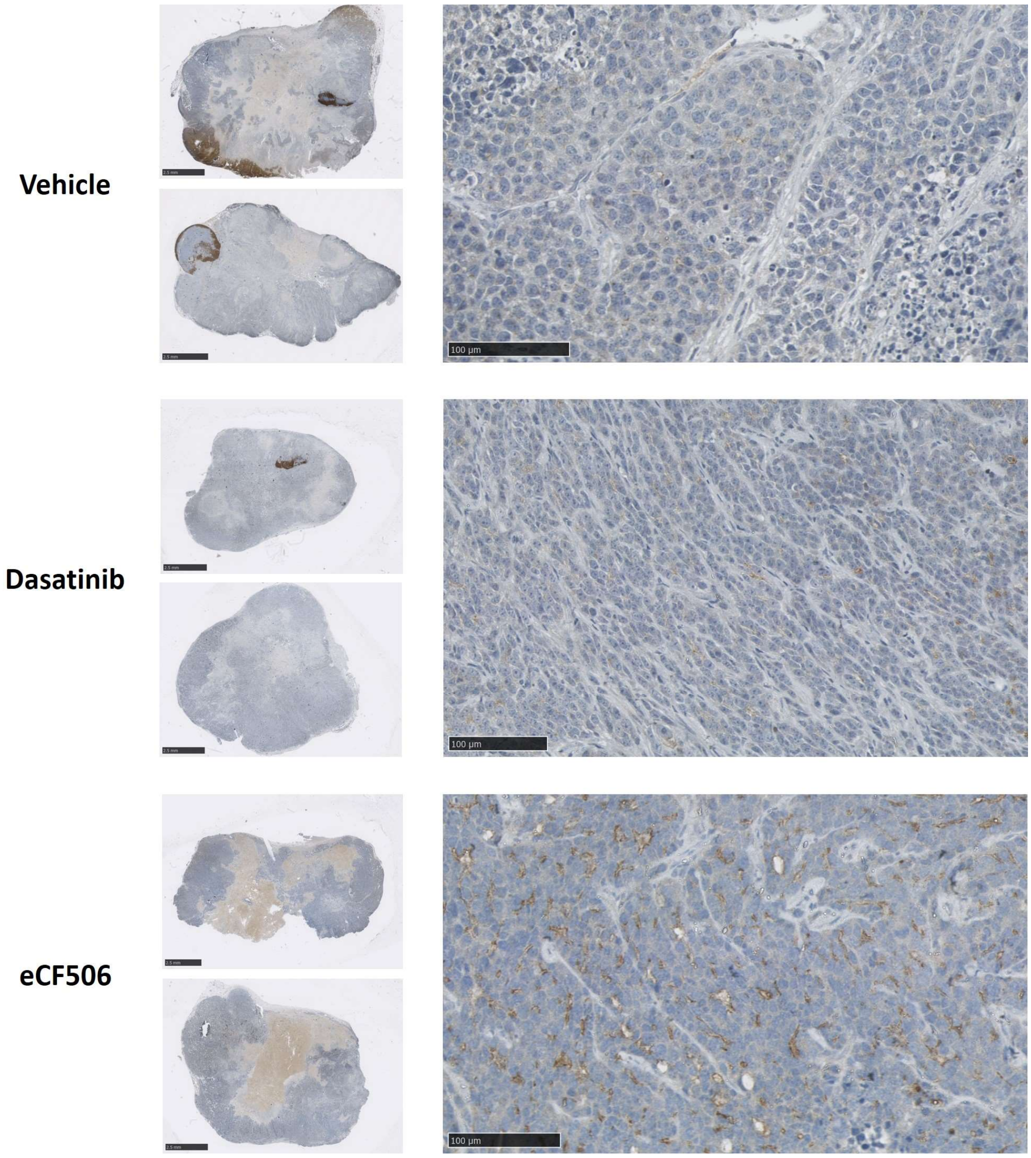

Figure S20. Representative IHC images of sections of MetBo2 orthotopic breast tumors treated with vehicle (left), dasatinib (centre) and eCF506 (right). Images illustrate two complete tumor sections and an amplification (20 x magnification) of the intratumoral area to identify CD3+ cells (brown). NOTE: tumors of mice treated with eCF506 did not show lymph node components (dark brown), while they were clearly observed in the tumor sections from mice treated with vehicle.

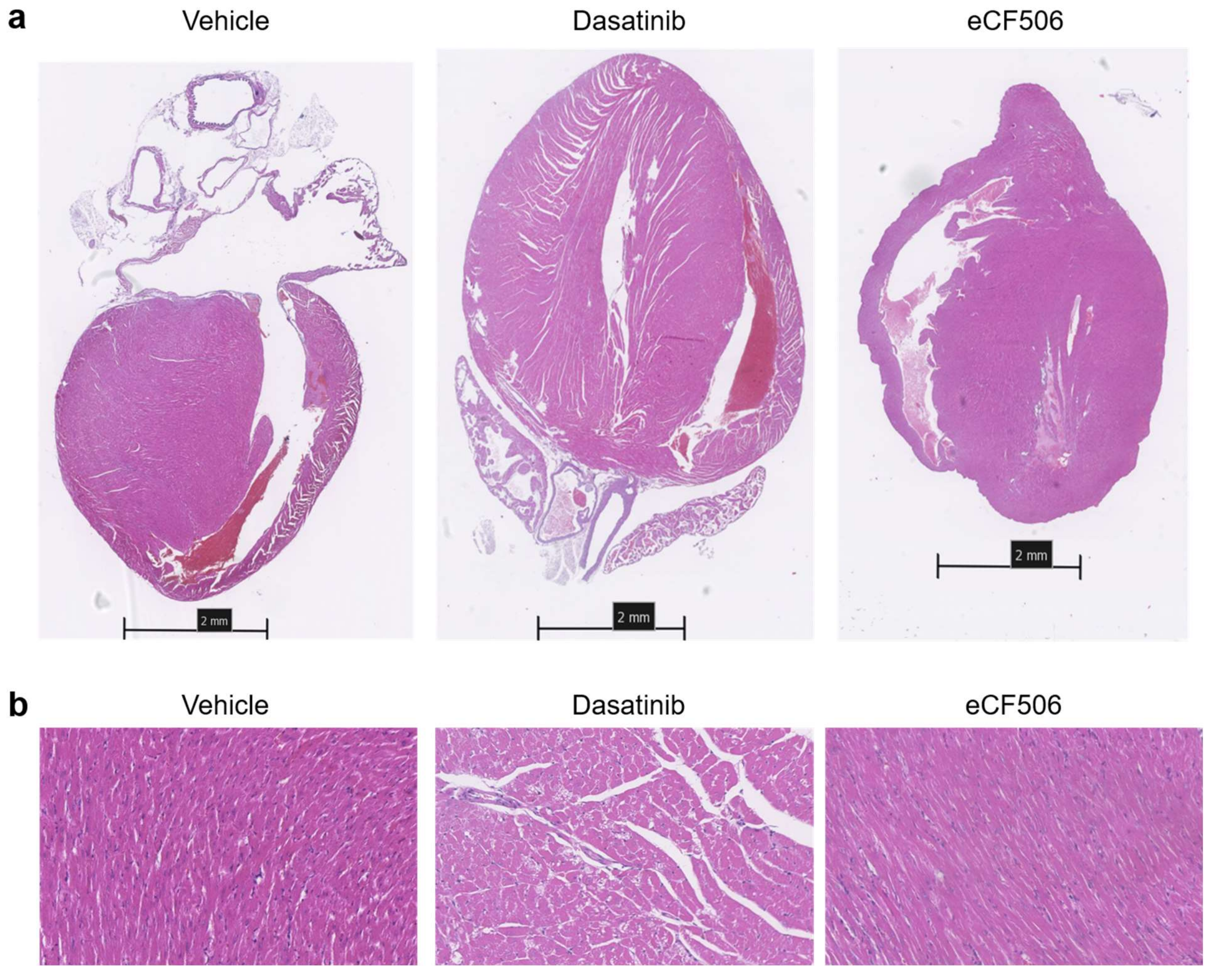

Figure S21. Representative H&E-stained histology images of hearts from tumor-bearing mice treated with vehicle (left), dasatinib (centre) and eCF506 (right). **(a)** Whole heart images (size bar 2mm). (b) Images (x20 magnification) of normal myocardium in the vehicle control and eCF506-treated murine hearts, but in dasatinib-treated heart there is focal myocyte hydropic change and variation in myocyte cell size, consistent with myocardial hypertrophy and focal cell injury.

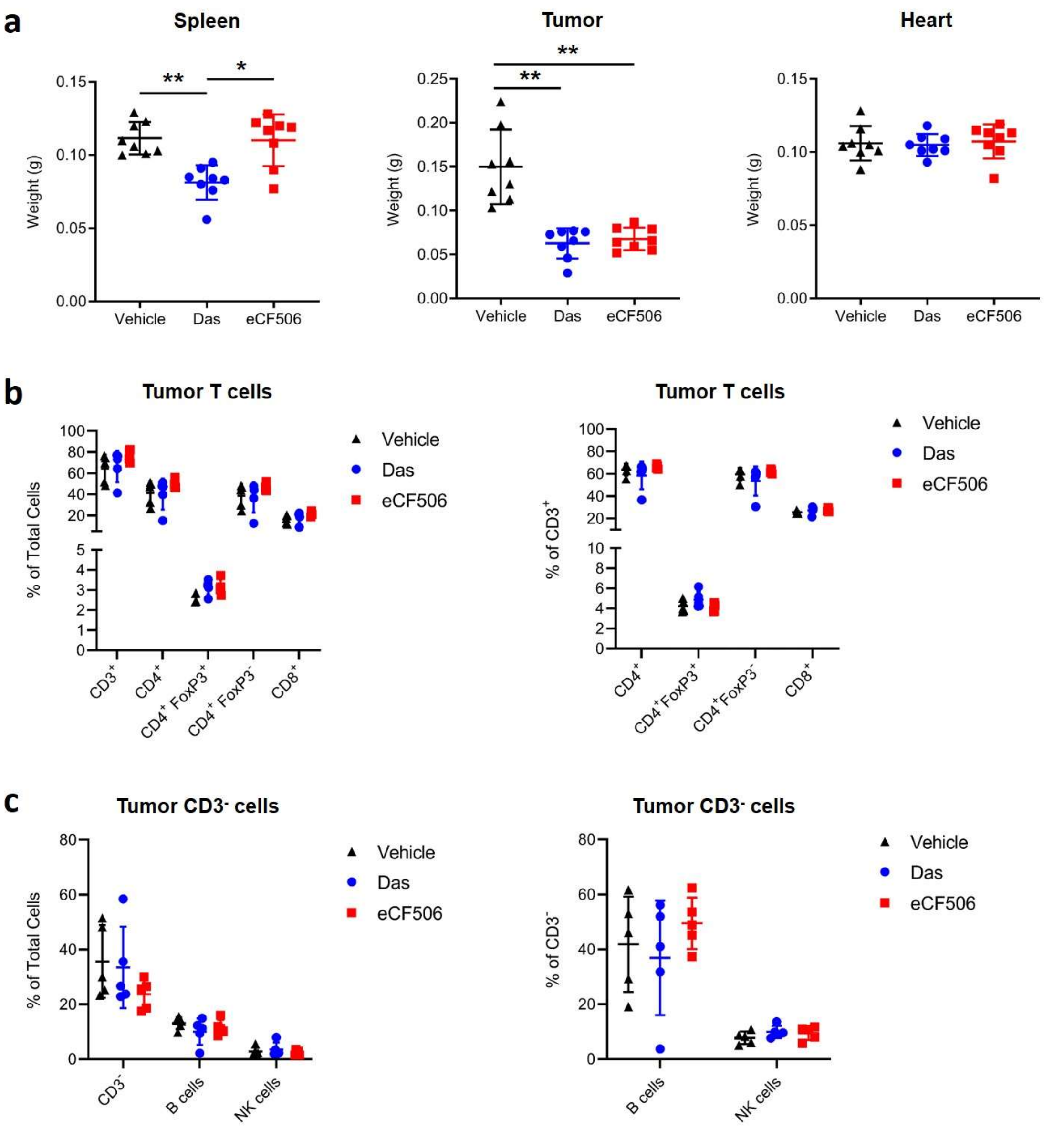

Figure S22. Weight analysis of organs and immune cell quantification of MetBo2 tumor samples from the 5-day treatment study. MetBo2 tumor bearing mice were treated with vehicle, dasatanib (Das) or eCF506 for 5 d, spleen, heart and tumor tissues harvested at endpoint. A) Spleen, tumour and heart weights, n=8 per group. Significance was assessed by t-test with * = <0.05, ** = <0.01. B,C) Immunophenotyping of tumor samples by flow cytometry, n=5 per group. B) Tumor infiltrating T cell subsets are shown as either a percentage of total cells (left) or CD3+ (right). C) Tumor infiltrating CD3- cell subsets are shown as either a percentage of total cells (left) or CD3- (right).

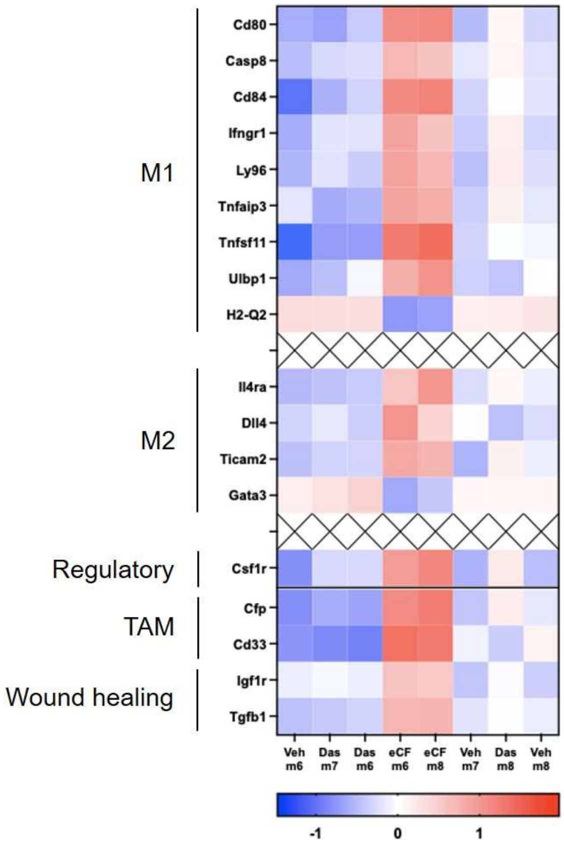

Figure S23. Transcriptomics analysis of tumor samples from the 5-day treatment study. RNA was extracted from tumor samples (n= 2 for eCF506-treated group, and n= 3 for vehicle- and dasatinib-treated animals) and analyzed using the PanCancer Immune Profiling panel in the NanoString nCounter® platform. Heatmap depicts individual pathway Z scores of greatest changes found from the gene expression screening of tumors treated with vehicle (Veh), dasatinib (Das) or eCF506 (eCF). mN identifies the origin of the sample.

References

1. Seeliger MA et al. High yield bacterial expression of active c-Abl and c-Src tyrosine kinases. *Protein Sci* **2005**;14:3135-39.

2. Levinson NM, Boxer SG. A conserved water-mediated hydrogen bond network defines bosutinib's kinase selectivity. *Nat Chem Biol* **2014**;10:127-32.

3. Kabsch WX. *Acta Crystallogr D Biol Crystallogr* **2010**;66:125-32.

4. Evans PR, Murshudov GN. How good are my data and what is the resolution? *Acta Crystallogr D Biol Crystallogr* **2013**;69:1204-14.

5. McCoy AJ et al. Phaser crystallographic software. *J Appl Crystallogr* **2007**;40:658-74.

6. Murshudov GN, Vagin AA, Dodson EJ. Refinement of macromolecular structures by the maximum-likelihood method. *Acta Crystallogr D Biol Crystallogr* **1997**;53:240-55.

7. Emsley P, Cowtan K. Coot: model-building tools for molecular graphics. *Acta Crystallogr D Biol Crystallogr* **2004**;60:2126-32.

8. Schuck P. Size-distribution analysis of macromolecules by sedimentation velocity ultracentrifugation and lamm equation modeling. *Biophys J* **2000**;78:1606-19.

9. Holde KE. *Physical biochemistry*. Pearson Prentice Hall, 1985.

10. Laue TM, Shah BD, Ridgeway TM, Pelletier SL. *Computer-Aided Interpretation of analytical sedimentation data for proteins.* Cambridge: Royal Society of Chemistry; 1992.

11. Jafari R *et al.* The cellular thermal shift assay for evaluating drug target interactions in cells. *Nat Protoc* **2014**;9:2100–22.

12. Anastassiadis *et al.* Comprehensive assay of kinase catalytic activity reveals features of kinase inhibitor selectivity. *Nat Biotechnol* **2011**;29:1039-45.

13. Macleod KG, et al. Reverse Phase Protein Arrays and Drug Discovery. *Methods Mol Biol* **2017**;1647:153–69.

14. Bankhead P, *et al.* QuPath: Open source software for digital pathology image analysis. *Sci Rep* **2017**;7:16878.

15. Nguyen et al. Identification of new binding proteins of focal adhesion kinase using immunoprecipitation and mass spectrometry. *Sci Rep* **2019**;9:12908.

16. Song et al. Dual inhibition of MET and SRC kinase activity as a combined targeting strategy for colon cancer. *Exp Ther Med* **2017**;14:1357.

17. Min et al. Targeting the insulin-like growth factor receptor and Src signaling network for the treatment of non-small cell lung cancer. *Mol Cancer* **2015**;14:113.

18. Hartman et al. The Tyrosine Phosphatase SHP2 Regulates Focal Adhesion Kinase to Promote EGF-Induced Lamellipodia Persistence and Cell Migration. *Mol Cancer Res* **2013**;11:651.

19. Zhang et al. Focal adhesion kinase promotes phospholipase C-gamma1 activity. *Proc Natl Acad Sci USA* **1999**;96:9021.

20. Giambelluca et al. Expression and regulation of glycogen synthase kinase 3 in human neutrophils. *Int J Biochem Cell Biol* **2013**;45:2660.

21. Marhäll et al. The Src family kinase LCK cooperates with oncogenic FLT3/ITD in cellular transformation. *Sci Rep* **2017**;7:13734.
